# Comparison of two carbohydrate-based biostimulant complexes for their ability to enhance *Cannabis sativa* flower yield and quality

**DOI:** 10.3389/fpls.2026.1842299

**Published:** 2026-06-05

**Authors:** Kimber Wise, Tomer Simovich, Reji Alengaden, Harsharn Gill, Adrian Zandberg, Jamie Selby-Pham

**Affiliations:** 1 School of Science, RMIT University, Melbourne, VIC, Australia; 2 Cannabis and Biostimulants Research Group, Melbourne, VIC, Australia; 3 School of Engineering, RMIT University, Melbourne, VIC, Australia; 4 PerkinElmer Inc., Melbourne, VIC, Australia; 5 Faculty of Science, Monash University, Melbourne, VIC, Australia; 6 Freelance Consultant, Melbourne, VIC, Australia

**Keywords:** *Aloe vera*, fish hydrolysate, galactooligosaccharide, molasses, odour, sensory, terpene, triacontanol

## Abstract

**Introduction:**

*Cannabis sativa* L. (cannabis) is valued for its flowers, which are rich in bioactive compounds that impart medicinal and sensory properties. A common-practice during plant cultivation is the supplementation of fertilisers with biostimulants, which enhance growth and yield. Previous studies on tomato, strawberry, capsicum, and cannabis (grown for seed) have demonstrated that biostimulants containing fish hydrolysate (FH), *Aloe vera* (L.) Burm.f. extract, molasses, or triacontanol can improve yield, quality, and functional food value. Additionally, galactooligosaccharides (GoS) are proposed as a novel biostimulant that selectively stimulates beneficial microbes, akin to a plant prebiotic.

**Methods:**

Two biostimulant complexes were compared by randomised controlled trial (RCT), within an environmentally controlled hydroponic growth system, for their effects on cannabis growth, yield, and flower quality. The two complexes tested were: BC1 comprising molasses, *A. vera* extract, and FH; and BC2 comprising GoS, *A. vera* extract, and triacontanol.

**Results and Discussion:**

Both treatments improved yield (BC1: 1.17-fold, p = 0.097; BC2: 2.22-fold, p = 0.003), with BC2 also increasing flower size (1.28-fold, p = 0.034). Image analyses indicated that neither treatment substantially impacted flower colour. Near infrared indicated that both treatments increased primary amines and methyl containing hydrocarbons, and that BC2 also increased aromatic hydrocarbons. Volatile analysis indicated that BC1 increased a-terpinolene, borneol, terpineol, and valencene, whilst BC2 increased a-humulene, a-phellandrene, α-terpinene, β-caryophyllene, guaiol, limonene, ocimene, and total terpene content. These terpene changes suggest possible increases to flower relaxing effects (BC1), or anti-inflammatory effects (BC2). Odour prediction identified possible shifts to flower odour profiles, which may be associated with enhanced customer perceptions of quality and value. Overall, this study demonstrates the potential for biostimulants to enhance cannabis yield, phytochemical composition, and associated value.

## 1 Introduction

*Cannabis sativa* L. (cannabis) is an environmentally-friendly, sustainable (Wise et al., 2023a), and economically important crop. Globally cannabis industries were evaluated at US$44 billion in 2022, and forecasted to grow up to US$444 billion by 2030 (Fortune Business Insights, 2024). Low-Δ-9 tetrahydrocannabinol (low-THC) cultivars (i.e. ‘hemp’) are grown for their fibre and seed which is a functional food, while medicinal-varieties (of varying THC levels) are grown for their flowers, which are rich in a range of therapeutically-relevant phytochemicals including the cannabinoids THC and cannabidiol (CBD), and volatile terpenes (Bonini et al., 2018; Duggan, 2021). Within the last decade, there has been an international trend towards legalisation of medicinal and recreational cannabis, which has facilitated increased access to legal cannabis cultivation, alongside increased ease of customer access to legal cannabis products (Steinberg, 2022). The increase in customer demand has imparted a positive pressure on the supply chain, resulting in the need for increased quantity and quality of cannabis production to fulfil market demands (Hansen et al., 2021). As such, there is growing interest in strategies to increase the cultivation efficiency and quality of cannabis products.

The quality of cannabis is largely attributed to the presence of secondary metabolites within the flowers, including volatiles, which impart therapeutic and sensory properties, and *in natura* function to protect the plant from stressors (Jan et al., 2021; Milay et al., 2020). One strategy for increasing these secondary metabolites in plants, is to use biostimulants, which are non-nutritive biological derivatives that have the capacity to enhance crop growth and yield, often via stimulation of stress-associated responses (du Jardin, 2015). Biostimulants are commonly used during crop cultivation, including cannabis; however, efficacies are species-specific and often have not been validated on cannabis within controlled studies (Bulgari et al., 2015). It is also common commercial practise for multiple biostimulants to be used in combination or applied as physically-combined complexes, which are thought to impart additive beneficial effects (Benito et al., 2022). However, these purported additive effects require validation, particularly their impacts on cannabis flower composition and quality.

For cannabis, induction of both biotic (Kostanda and Khatib, 2022) and abiotic (Park et al., 2022) stressors may modulate volatiles production (Reichel et al., 2022). Since induction of stress responses is a common effect attributed biostimulants, supplementation of fertiliser with biostimulants is a potential strategy for the enhancement of cannabis flower volatiles. Accordingly, the work herein explored the potential for two biostimulant complexes (BCs) to impact cannabis yields; BC1 comprising molasses, *Aloe vera* (L.) Burm.f. extract, and fish hydrolysate (FH), and BC2 comprising galactooligosaccharides (GoS), *A. vera* extract, and triacontanol (TRIA), with background to these compositions presented below.

*A. vera* extract, fish hydrolysate (FH), and molasses are all naturally derived biostimulants that have been shown to impact plant growth and stress responses through distinct mechanisms. *A. vera* leaf extract contains a range of bioactive compounds, including macro- and micro-nutrients, vitamins, enzymes, amino acids, sugars, plant sterols, auxins, gibberellins, and salicylic acid (Chatterjee et al., 2013; El Sherif, 2017), and has been shown to enhance growth and yield across several crops including lavender (El Sherif, 2017), okra (Hemalatha et al., 2018a, 2018b), sweet basil (Hamouda et al., 2012) and caraway (Khater et al., 2020). Fish hydrolysate is derived from fish products and contains predominantly amino acids and short peptide fragments (Colla et al., 2015), and has been shown to benefit growth, yield, nutrient uptake, and phytochemical composition in numerous fruit and vegetable crops (Colla et al., 2015; Halpern et al., 2015; Wise and Selby-Pham, 2023). Molasses is a by-product of sugar production from sugarcane or beetroot, containing simple carbohydrates such as sucrose, glucose, and fructose (Chandra et al., 2008; Ito, 1976; Mee et al., 1979; Najafpour and Shan, 2003; Palmonari et al., 2020), and when applied as a biostimulant has been observed to increase the antioxidant capacity of hemp seeds (Wise et al., 2024c) and reduce cannabis flower terpene concentrations (Wise and Selby-Pham, 2026b). The modes of effect of each of these biostimulants remains an ongoing area of research, however, elicitation of plant stress responses appears to be a shared effect. In young cannabis plants, application of *A. vera* extract was shown to induce general stress mechanisms, while FH induced pathogen-associated mechanisms (Wise et al., 2024a). It was suggested that the effect from FH might relate to trace amounts of chitin, which might be present if crustacean bycatch is included with fish material during processing. Molasses is hypothesised to elicit plant stress-responses through two mechanisms, 1) direct stimulation by the exogenous detection of endogenous cellular compounds, such as sugars, and 2) indirect stimulation by the promotion of microbial growth within the rhizosphere, against which plants may then mount a defence response (Waguespack et al., 2022; Wise et al., 2024c). Accordingly, each of these biostimulants are of interest to cannabis cultivation for their potential to modulate secondary metabolites through elicitation of the plant stress response.

In recent studies, a biostimulant complex comprising molasses, *A. vera* extract, and FH (herein referred to as ‘BC1’), demonstrated improvements to the quantity, quality, or functional food potential of: tomato (Wise and Selby-Pham, 2025a), strawberry (Wise and Selby-Pham, 2025b; Wise et al., 2024b), and hemp seed (Wise et al., 2025). These prior studies indicate the capacity of BC1 to impact plant primary and secondary metabolism and thereby provide substantial value to cultivators. Furthermore, Wise et al. (2025b) identified that fertiliser supplementation with BC1 induced a salicylic acid (SA)-driven (microbially-associated stress response) which resulted in increased THC content but decreased volatile contents in pollenated industrial cannabis (hemp) flowers (*C. sativa* cv. Fairnsfield). Accordingly, application of BC1 to a feminised cannabis cultivar (cv. CBG Force) was explored within the present study, to characterise anticipated biostimulant effects of BC1 during cultivation of medicinal cannabis.

The other BC tested (herein referred to as ‘BC2’) also contained *A. vera* extract, as well as TRIA and GoS. Triacontanol (TRIA) is a saturated primary alcohol that was originally isolated from alfalfa, and has since been identified as a plant growth regulator (PGR) capable of promoting growth, yield, and stress resistance of crops (Sharma and Kapoor, 2023; Verma et al., 2022). Fertiliser supplementation with TRIA, or TRIA-containing complexes, has been shown to enhance the yield of tomato (Borowski et al., 2000; Khan et al., 2009), strawberry (Tiwari et al., 2017), and capsicum (Wise et al., 2023c). Additionally, in a recent study, (Wise and Selby-Pham, 2026a) observed that provision of a TRIA-containing BC to cannabis enhanced flower yield. Transcriptomic analyses of strawberry (Pang et al., 2020) and rice (Chen et al., 2002) identified that a mode of effect of exogenous TRIA application was modulation of gene expression associated with primary metabolism as well as genes associated with stress mechanisms.

By contrast to the simple sugars abundant in molasses, galactooligosaccharides (GoS) are complex sugars, which are utilised as dietary supplements for humans to selectively promote beneficial gut microbes (Davani-Davari et al., 2019; Sangwan et al., 2011). The capacity for microbes, either within the gut or within rhizospheres, to utilise GoS depends on their ability to produce galactosidases, which are the enzymes required to hydrolyse these complex sugars and utilise the carbon for energy (Belorkar and Gupta, 2016). Whilst these enzymes are present in some PGPRs and the beneficial fungi *Trichoderma* (Danilovic et al., 2016), the associated genes are not found in the genomes of oomycetes, which are a common pest in hydroponic systems (Zerillo et al., 2013). Accordingly, as the biostimulant potential of carbohydrate sources is tied to provision of microbially-available carbon, the selectivity of GOS (by contrast to simple sugars) positions it as a potentially more desirable option. This is supported by recant results within Wise and Selby-Pham (2026b), wherein GOS (but not molasses) impacted cannabis flower odour, with the modulations predicted to enhance hedonic quality assessments.

## 2 Materials and methods

### 2.1 Materials

The biostimulant complexes (BCs) utilised were: biostimulant complex 1 (BC1), which was an aqueous solution containing 7% v/v (10% w/v) molasses, 2.5% v/v *A. vera* extract, and 5% v/v fish hydrolysate (FH); and biostimulant complex 2 (BC2), which was an aqueous solution containing 1.25% w/v galactooligosaccharides (GoS), 2.5% v/v *A. vera* extract, and 0.25 ppm triacontanol, both of which were generously provided by Nutrifield Pty Ltd (Melbourne, VIC, Australia).

### 2.2 Propagation, plant growth, and flower harvest

All plants utilised in experiments were low-THC, high-cannabigerol (high-CBG) female *C. sativa* plants (cv. CBG Force). Propagation was performed by taking vegetative cuttings from a single stock plant, dipping them into a rooting hormone (Clonex Purple, Growth Technology, O’Connor, WA) and placing them inside aeroponic mist-propagation units (EzClone Aeroponic Classic Cutting System - 16, EZCLONE Enterprises Inc., Sacramento, CA) containing hydroponic nutrients (Coco A&B, Nutrifield Pty Ltd, Melbourne, VIC, Australia) prepared to an electrical conductivity (EC) of 0.8, and within an environmentally controlled growth room, as described in Wise et al. (2020). Briefly, the conditions during propagation were as follows: the light:dark (L:D) ratio was 18:6, the day temperature was 22 °C, the day relative humidity (RH) was 70%, the night temperature was 19 °C, and the night RH was 51%. After approximately two weeks, 18 mature cuttings (exhibiting branched root systems) of consistent size, were planted into individual 27L pots with a coir perlite substrate (Coco Perlite premium pure blend 70/30, Nutrifield Pty Ltd, Melbourne, VIC, Australia), and transferred into the experimental growth room with artificial lighting and environmental conditions as described in Wise et al. (2025a).

For 4 weeks the plants received a L:D ratio of 18:6 (vegetative period), and then for 8 weeks the L:D was 12:12 (flowering period). During the lighting period the temperature was 27 °C and the RH was 70%, and during the dark period the temperature was 21 °C and the RH was 50%. Fertigation was prepared weekly, using commercial hydroponic nutrients (Coco A&B, Nutrifield Pty Ltd, Melbourne, VIC, Australia), and provided to plants daily via an automated drip irrigation system, as described in Wise et al. (2025a). Details on the weekly strength and volume of the fertigation that was provided to plants are presented in **Supplementary Tables S1**, **S2**.

The biostimulant treatments were provided to the plants during weeks 5–12 by addition into the respective reservoirs. Six plants (n = 6) received 2 ml/L of BC1, 6 plants received 2 ml/L of BC2, and 6 plants were the control set that did not receive anything additional to their fertigation solutions. After 12 weeks, stem width was measured using callipers (Digital Vernier Calliper 150 mm, Kincrome, Scoresby, VIC, Australia), and the plants were then cut at the base, measured for fresh weight (FW) biomass, and hung upside down within the grow room for two weeks to dry in the dark, at 18 °C, and 50% relative humidity (RH). After two weeks, inflorescences (flowers) were harvested, processed, and cured, including weighing of the cured flower yield (CW), as per Wise et al. (2025b). The flowers from each plant were bulked together for curing within commercial curing bags (TerpLoc, Grove Bags LLC, Bedford Heights, OH, USA) for 1 week in the dark at 25 °C. Flowers were taken from this bulked yield at random for further analyses (described below).

### 2.3 Flower dimensions and colour analyses

Flower dimensions and colour analysis were measured on the cured flowers. Individual flower ‘size’ was defined as the flower’s longest diameter, which was measured using digital callipers. Flower size was categorized using an adaptation of the criteria presented by Harvest Hub (2022) (https://www.twistertrimmer.com/cannabis-size-sorting/), where flowers were categorised as ‘Grade A’ if their size was greater than 15.875 mm, ‘Grade B’ if between 12.700–15.875 mm, and ‘Shake’ if smaller than 12.700 mm.

Flower area and colour distribution were measured by image analysis, using the method first presented in Wise et al. (2022) with adaptations presented in Wise et al. (2025a). As per these methods, flower images were collected in a standardised manner: briefly, this involved placing 22–33 flowers (one image of flowers per plant) on a white A4 piece of paper containing a reference square of 9 cm^2^, on an assigned bench within the laboratory for consistency of lighting, and photographed using a consistent camera from a distance of approximately 30 cm. Prior to analysis, the images were pre-processed in Microsoft Paint using ‘Remove Background’ and ‘Fill’ tools to colour the background cyan (RGB 0:255:255) and the reference square purple (RGB 255:0:255). Flower ‘area’ was calculated as the total pixel count minus cyan (background) and purple (reference square), divided by the number of flowers within the image, followed by conversion from pixels to area (based on the total purple pixels representing 9 cm²).

The image colour analysis workflow utilised in this study was based on the method described in Wise et al. (2022), adapted to achieve colour categorisation based on CIE L*a*b* (Lab) space rather than RGB. This was achieved using a script coded in Python (Appendix I), which converts the image RGB data into Lab colour data, and then categorises the pixels using a rule-based colour classification method, adapted from Benavente et al. (2008). For the colour classification, each pixel is assigned to an achromatic or near-white class based on explicit thresholds for lightness (L), and chroma (C) which is computed as $C=\;\sqrt{a^{2}+b^{2}}$. Pixels with very low chroma (C ≤ 4.0) are treated as achromatic and classified as black (L< 28.28), grey (28.28 ≤ L< 79.65), or white (L ≥ 79.65). In addition, pixels are also classified as white if they fall within a near-white or highlight region defined by either high lightness and low chroma (L ≥ 79.65 and C ≤ 9.0) or extreme highlights (L ≥ 97.0 and C ≤ 16.0), ensuring robust handling of specular and overexposed areas. Pixels that do not fall within these L and C thresholds (chromatic pixels) are categorised into one of nine colours (**Supplementary Table S3**), based on their hue-sector criteria defined within six lightness bands (L1 “very dark”:<20, L2 “dark”: 20–35, L3 “mid-dark”: 35–50, L4 “mid”: 50–65, L5 “mid-light”65–80, and L6 “light”: ≥80) and their Hue angle (h), which is derived from the a/b components using an arctangent transformation (h = atan2(b, a); Equation 1) mapped to 0–360°.

(1) $$atan2\left(b,a\right)=\{\begin{array}{c} arctan\left(\frac{b}{a}\right),\;a\;>\;0\\ arctan\left(\frac{b}{a}\right)\;+\;\pi ,\;a\;<0,\;b\geq 0\\ arctan\left(\frac{b}{a}\right)\;-\;\pi ,\;a\;<0,\;b<0\\ +\frac{\pi }{2},\;a=\;0,\;b\;>\;0\\ -\frac{\pi }{2},\;a=\;0,\;b\;<\;0 \end{array}$$

As colour may be considered as occurring within ‘fuzzy colour spaces’ (Mengíbar-Rodríguez and Chamorro-Martínez, 2022), the hue sectors were intentionally defined with partial overlap to accommodate instability in hue estimates arising from noise, low chroma, and reduced lightness, particularly in darker regions of the image. Classification is resolved through an explicit hierarchical evaluation in which overlapping sectors are tested in a fixed, predefined order, with earlier sectors taking precedence; this ordering was selected to bias ambiguous hues toward perceptually and empirically more plausible colour classes (e.g. green over cyan at low lightness), thereby improving classification stability without imposing rigid, non-overlapping hue boundaries. Within each lightness band, the chromatic hierarchy is evaluated in the following order: pink, red, brown, orange, yellow, green, cyan, blue, and then purple. Cyan (background) and purple (reference square) were excluded from the flower image colour analysis.

### 2.4 Flower chemical analyses

Near-infrared (N-IR) spectrometry of 1 cured flower per plant (n = 6) was performed as per Wise et al. (2025b) with a minor variation, that spectra were collected with a 2 cm^-1^ resolution over the 4,000–10,000 cm^-1^ (1.0–2.5 µm) range. The N-IR spectra were used for cannabinoid (CBD and THC) quantification in cured flowers as per Wise et al. (2025b). Additionally, headspace gas chromatography mass spectrometry (HS-GC/MS) of 1 cured flower per plant (n = 6) was performed as per Wise and Selby-Pham (2026b) to quantify: α-humulene, α-phellandrene, α-pinene, α-terpinene, α-terpinolene, β-caryophyllene, β-myrcene, β-pinene, bisabolol, borneol, camphene, eucalyptol, fenchol, fenchone, γ-terpinene, guaiol, limonene, linalool, ocimene, p-cymene, terpineol, and valencene. Following, the quantitative HS profiles were converted into odour intensity (OI) profiles by vector modelling as per Wise et al. (2023b), with a minor adaptation that for concentrations below a compounds odour detection threshold (ODT), the associated contribution to OI from that compound was assigned as 0, and K values from Goodscents were updated (sourced July 2025).

### 2.5 Data analyses

Treatment effects were assessed compared to control via 2-sample *t*-test, performed in Minitab 21.4 statistical software package (Minitab Inc., State College, PA), with the exception of measures that were not detected in control, which were instead analysed for statistical deviation from 0 by 1-sample *t*-test. Treatment impacts to flower size were assessed in Minitab by general linear model (GLM), with ‘plant’ as a random factor nested within ‘treatment’. Comparison of treatment impacts to flower grading probabilities was assessed by ordinal logistic regression using the Numiqo online platform (https://numiqo.com/statistics-calculator/regression, Accessed May 2026). Comparison of treatment impacts to flower grading proportions was assessed in Minitab by 2-proportions test, and Statistics Kingdom online calculator (https://www.statskingdom.com/proportion-confidence-interval-calculator.html) was used to determine 95% confidence intervals (CI), both of which utilised normal approximations. Near-infrared data was standardised across all samples to 9044 cm^-1^, with values below 0 assigned as 0 absorbance for that wavenumber.

All profile analyses were performed using the web-tool Metaboanalyst 6.0 (Chong et al., 2019). For flower colour, N-IR spectra, terpenes, and OI data heatmap and principal component analysis (PCA) were utilised. For profile analyses, normalisation was achieved for each dataset by the following transformations and scaling within Metaboanalyst: for colour profiles, log base 2 transformation and auto scaling; for HS profiles, no transformation and auto scaling; for OI profiles, cube root transformation and range scaling; and for N-IR profiles, log transformation and Pareto scaling. During PCA, statistical comparison of profile centroids was assessed by PERMANOVA within Metaboanalyst. For heatmap assessment, Euclidian distance measure and Ward clustering methods were utilised. During analysis of N-IR data within the Metaboanalyst platform, ANOVA was also utilised to identify regions of wavenumbers different between treatments.

To explore how OI changes might influence customer impressions, statistical modelling was conducted on OIs that were impacted (with significance or marginal significance) by at least one BC treatment and present within the sourced datasets. These sourced data sets were comprised of paired data of customer impressions of cannabis flowers with perception of odour descriptors (ODs) or OIs. The first dataset, sourced from Gilbert and DiVerdi (2018), included paired data (n = 793) on customer scoring for the presence of ODs, alongside scores for the customers’ interest in consuming the product (‘interest’) and the price they would be willing to pay per gram (‘price’). For this data set, ‘interest’ and ‘price’ of samples scored for presence of the ODs, were compared to samples scored for the absence of the OD by 2-sample *t*-test in Minitab. The second data set, sourced from Wise et al. (2025b), included paired data (n = 84) on customer scoring of OI along with scored ‘quality’ and ‘desirability’. For this data set, odours were considered as ‘detected’ if they were scored as at least 1 (“mild odour”), flowers were considered as ‘desirable’ if desirability was scored as at least 1, and as ‘high quality’ if quality was scored as at least 1. The impact of OD detection to the assignment of samples as desirable or of high quality was assessed by 2-proportions test in Minitab. Additionally, ordinal logistic regression was performed utilising the Numiqo online platform (https://numiqo.com/statistics-calculator/regression, Accessed October 2025), to explore the impacts of detection of multiple ODs on the likelihood of flowers being assessed as desirable or high quality.

**Figure 1:**
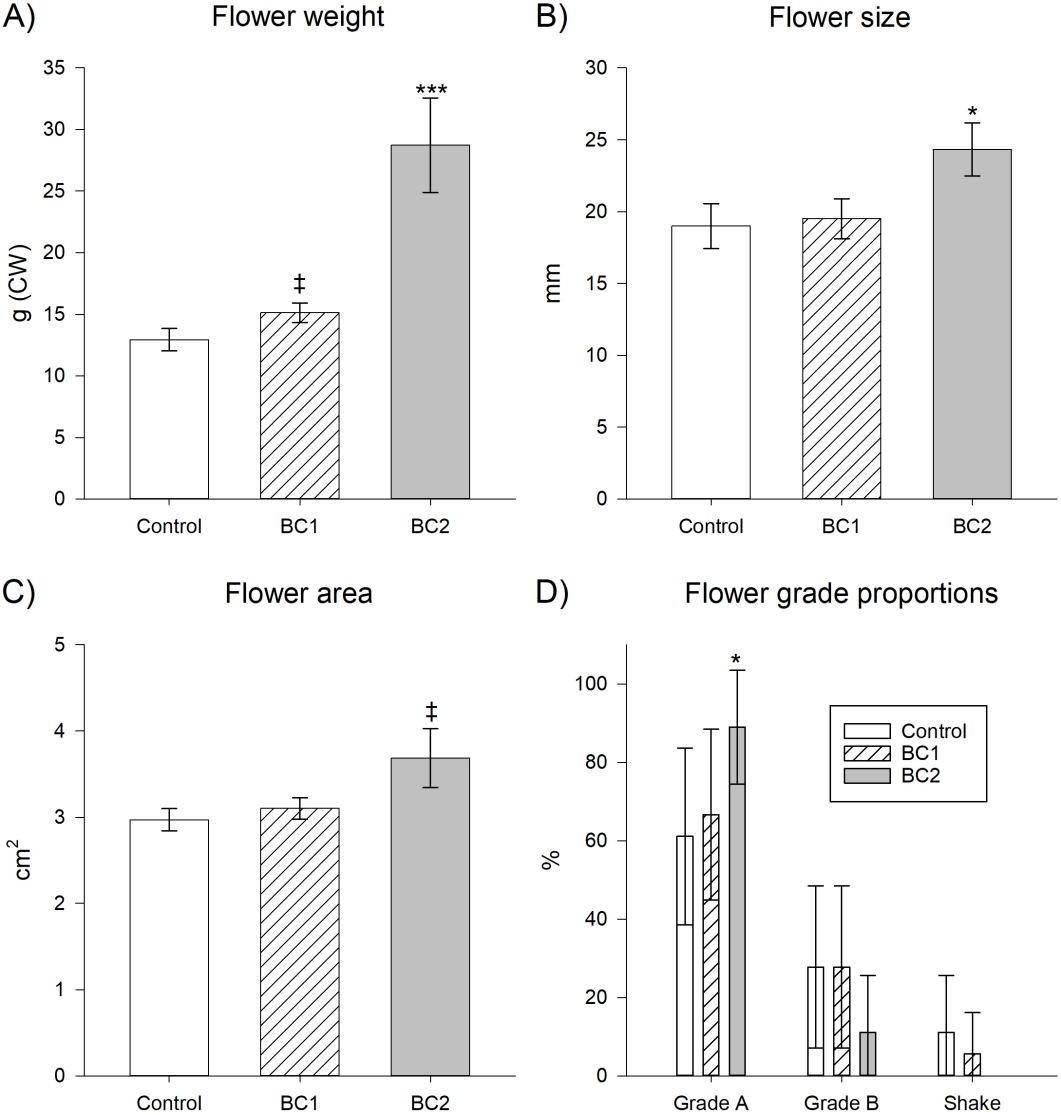
Impact of biostimulant complexes (BCs) to yield and size of cannabis flowers. Average measures of **(A)** flower yield (6 plants per treatment), **(B)** flower size (18 flowers per treatment), **(C)** Average flower area per plant 6 plants per treatment, and **(D)** flower grade proportions (18 flowers per treatment) from cannabis plants grown with nutrients supplemented with either BC1 or BC2. **(A–C)** Data presented as the mean ± standard error, **(D)** data presented as the mean proportions with error bars indicating the 95% CI. **(A–C)** Statistical significance calculated by 2-sample *t*-test compared to the control, **(D)** statistical significance calculated by 2-proportions test compared to control, and indicated by *** for p< 0.001, * for p< 0.05 and marginal significance indicated by ‡ for p< 0.1.

To explore implications of treatment-associated changes to N-IR spectra, functional group absorption wavenumbers were extracted from **Figure 1** (“Absorption Bands in the Near Infrared”) within Harris and Altaner (2013) using the online webtool WebPlotDigitizer (https://automeris.io/WebPlotDigitizer.html) to determine and assign spectral regions that were indicative of compounds containing the associated functional group. To estimate the relative abundance of compounds containing the associated functional groups, absorbances were summed across their associated spectra regions. The summed absorbance values were then analysed by 2-sample *t*-test in Minitab to explore whether the BC treatment impacted flower levels of compounds containing the associated functional group.

## 3 Results and discussion

Neither BC treatment was observed to impact plant vegetative growth, as indicated by an absence of significant differences to stem thickness or plant biomass (**Supplementary Table S4**). However, both treatments resulted in on-average increases to yield (**Figure 1A**), which included a marginally significant 1.17-fold increase for BC1 (p = 0.097), and a significant 2.22-fold increase for BC2 (p = 0.003). Flower yield is the primary metric of cannabis productivity (Dang et al., 2022) and profitability (Eaves et al., 2020), and as such, strategies to increase flower yield (such as via fertiliser supplementation) fulfil a key goal of cannabis cultivation research (Backer et al., 2019). Accordingly, the results from this preliminary study with 6 plants, demonstrate that biostimulants have the potential to increase cannabis yields. In particular, that inclusion of BC2 within fertigation regimens may provide cultivators with a strategy to substantially enhance operational efficiency and profitability via yield increases, which should be validated in follow-on large scale plant trials.

Exploration of the impacts to flower dimensions identified that BC2 resulted in a significant 1.28-fold increase to flower size (p = 0.003) and a marginally significant 1.24-fold increase to flower area (p = 0.098), whereas BC1 did not significantly impact either flower size or flower area (**Figures 1B, C**; **Supplementary Table S5**). When comparing flower size by GLM, ‘plant’ as a nested random factor within treatment was not significant for either BC1 (p = 0.995) or BC2 (p = 0.983), and so was not considered as a potential confounding factor in flower grading comparisons. Since harvested flowers were bulked prior to curing, all flower analyses were performed on random flower samples and did not account for potential positional variation within the plant, which is known to influence flower size (Naim-Feil et al., 2022) and composition (Namdar et al., 2018). However, this random sampling approach may better reflect representative flower material typically present in commercial post-harvest batches.

Ordinal logistic regression identified that BC1 did not significantly impact the probability of flowers being graded as either Shake (p = 0.543), Grade B (p = 1.000), or Grade A (p = 0.729), and BC2 did not significantly impact the probability of flowers being graded as either Shake (p = 0.089) or Grade B (p = 0.200). However, a significant model (p = 0.049) for probability of Grade A assignment was achieved for BC2 flowers, with the BC2 treatment having a positive coefficient of 1.63 (p = 0.068), and an odds ratio of 5.09. Accordingly, the ordinal logistic regression indicated that BC2, but not BC1, was associated with a beneficial shift in flower grade probabilities, specifically through an increased likelihood to flowers being categorised as Grade A. This change in probability was reflected by a 1.45-fold increase from 61.11% Grade A for control to 88.69% for BC2, which was significant by 2-proportions test (p = 0.042). This increase in Grade A flowers may reflect a shift in the overall quality profile of flowers produced by BC2, with the proportions of lower-grade flowers also tending to be reduced. Specifically, Grade B proportions were reduced by 0.40-fold, from 27.78% for control to 11.11% for BC2 (p = 0.196), while reductions in the lowest grade, Shake, were even more apparent, with Shake accounting for 11.11% of control flowers but 0% for BC2 flowers (p = 0.134) (**Figure 1D**; **Supplementary Table S6**).

Flower size is a driver of cannabis flower value, as customers perceive larger flowers as higher-quality, which therefore impacts the likelihood to purchase (Belackova, 2020; Canxchange, 2024; Huff et al., 2021). Accordingly, flowers fulfilling a sufficient size criteria may be sold as high-value intact flowers, while smaller flowers, deformed flowers, or flower fragments tend to be sold at a lower value, or are utilised for extraction prior to sale (Rosenthal, 2017). Grade A flowers sell for approximately 3.6-fold the value of Grade B flowers, which in-turn sell for approximately 5-fold the value of the lowest grade Shake flowers (Harvest Hub, 2022). Accordingly, based on the change in each grade proportion, the BC2 treatment resulted in a 1.32-fold increase in flower value per unit weight. When projecting this change in proportions onto the measured 2.22-fold increase in flower yield, the combined effect corresponds to an overall 2.93-fold increase to yield value for BC2. By contrast, the BC1 treatment resulted in a 1.08-fold increase in flower value per unit weight based on the grade proportions, which when projected onto the 1.17-fold increase to measured yield, corresponds with an overall 1.26-fold increase to harvest value. Accordingly, BC2 substantially outperformed BC1 in terms of capacity to increase yield value via increases to size grading in addition the impacts to yield mass, mentioned above.

In addition to flower size, flower quality is also impacted by flower colour (Donnan et al., 2022). Accordingly, image analysis was utilised to explore BC-treatment associated changes to cured flower colour. Principal component analysis (PCA) indicated that the colour profiles of the 3 treatment groups were not distinct (overlapping of all three 95% CI; **Supplementary Figure S1**) and pair-wise PERMANOVA results indicated no significant difference between the BC2 and control centroids (p = 0.172), nor significant difference between the BC1 and control centroids (p = 0.613). Accordingly, neither the BC2 treatment nor the BC1 treatment, appeared to result in a shift to the average flower colour profile that is distinct from the control flower colour profile. In addition to these colour profile analyses, individual colours were also compared between treatments. Blue was not detected in any sample, additionally brown and pink accounted for 0.002–0.007%, and as such blue, brown, and pink were removed from further analyses (**Supplementary Table S7**). Whilst the BC1 treatment did not result in any significant changes to individual flower colours, the BC2 treatment was associated with a significant decrease to grey (0.53-fold decrease, p = 0.014) and a marginally significant decrease to orange (0.76-fold decrease, p = 0.085). Additionally, whilst not statistically significant, on-average BC2 was associated with a 0.82-fold reduction in red (p = 0.126), a 1.01-fold increase in green (p = 0.372), and a 1.32-fold increase in black (p = 0.189). As such, on average, the overall impact of the BC2 treatment may be represented as arithmetic shifts (of total assigned flower image pixels): 0.3% increase to black, 1.2% reduction to grey, 0.1% reduction to orange, 0.1% reduction to red, and a 1.1% increase to green. However, noting the small treatment-associated effect sizes, these colour shifts are not anticipated to impact customer impressions of the cannabis flowers.

**Figure 2:**
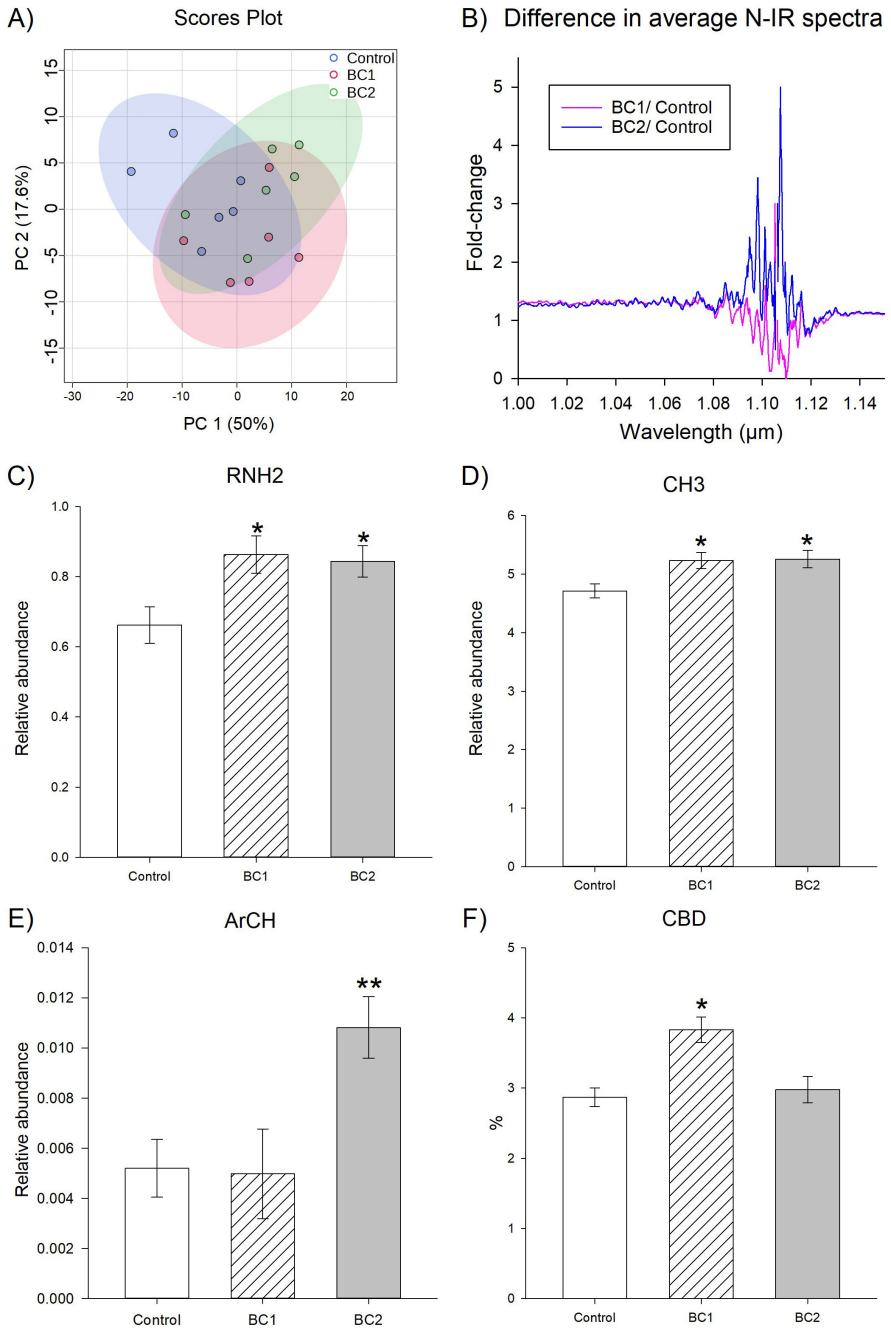
Impact of biostimulant complexes (BCs) to cannabis flower molecular composition determined by N-IR analyses. **(A)** Principal component analysis (PCA) of the N-IR spectral profiles from 6 flowers per treatment, with shading indicating 95% confidence intervals (CI) (blue: control, red: BC1, green: BC2). **(B)** Average near-infrared (N-IR) spectra for a region of interest between 1.00–1.15µm. Relative abundance of **(C)** primary amines, **(D)** methyl group-containing hydrocarbons, and **(E)** aromatic hydrocarbons, inferred by total absorbance over their associated spectra regions. **(F)** CBD abundance. **(C–F)** Data presented as the mean ± standard error, with statistical significance indicated by ** for p< 0.01, and * for p< 0.05, calculated by 2-sample *t*-test compared to control.

Flower scans by N-IR indicated that overall the spectral profiles were not significantly changed, as indicated by overlapping 95% CI during PCA (**Figures 2A, B**). However, pair-wise PERMANOVA results indicated that there was a significant difference between the BC1 and control centroids (p = 0.027) and between the BC2 and control centroids (p = 0.037). Within Metaboanalyst, ANOVA identified the 1.03–1.14 µm region as significantly different between treatments (p< 0.01). Within this region of interest, absorption bands associated with functional groups were identified from Harris and Altaner (2013), which included: primary amines over the 1.017–1.043 µm region, aromatic hydrocarbons over the 1.094–1.099 µm region, and methyl group-containing hydrocarbons over the 1.118–1.194 µm region (**Supplementary Table S8**). Based on the changes in these spectral regions associated with each of these functional groups, it appears that both treatments were associated with a significant increase to primary amines (**Figure 2C**; BC1: 1.30-fold increase, p = 0.023; BC2: 1.27-fold increase, p = 0.025), and methyl group-containing hydrocarbons (**Figure 2D**; BC1: 1.11-fold increase, p = 0.017; BC2: 1.12-fold increase, p = 0.017). However, while BC2 appeared to increase aromatic hydrocarbons (2.08-fold increase; p = 0.007), this region was not significantly (p = 0.927) impacted by BC1 (**Figure 2E**). Analysis of the IR spectra for cannabinoid quantification (**Supplementary Table S9**) indicated that THC was below the limit of detection (LoD) for all flower samples analysed, and that whilst BC2 did not significantly impact CBD (p = 0.642), BC1 significantly (p = 0.002) increased CBD by 1.33-fold (**Figure 2F**). These IR results, which were based on six plants per treatment, represent preliminary evidence that both BC treatments increased primary amines and methyl group-containing hydrocarbons, that BC2 (but not BC1) increased aromatic hydrocarbons, and that BC1 (but not BC2) increased CBD.

**Figure 3:**
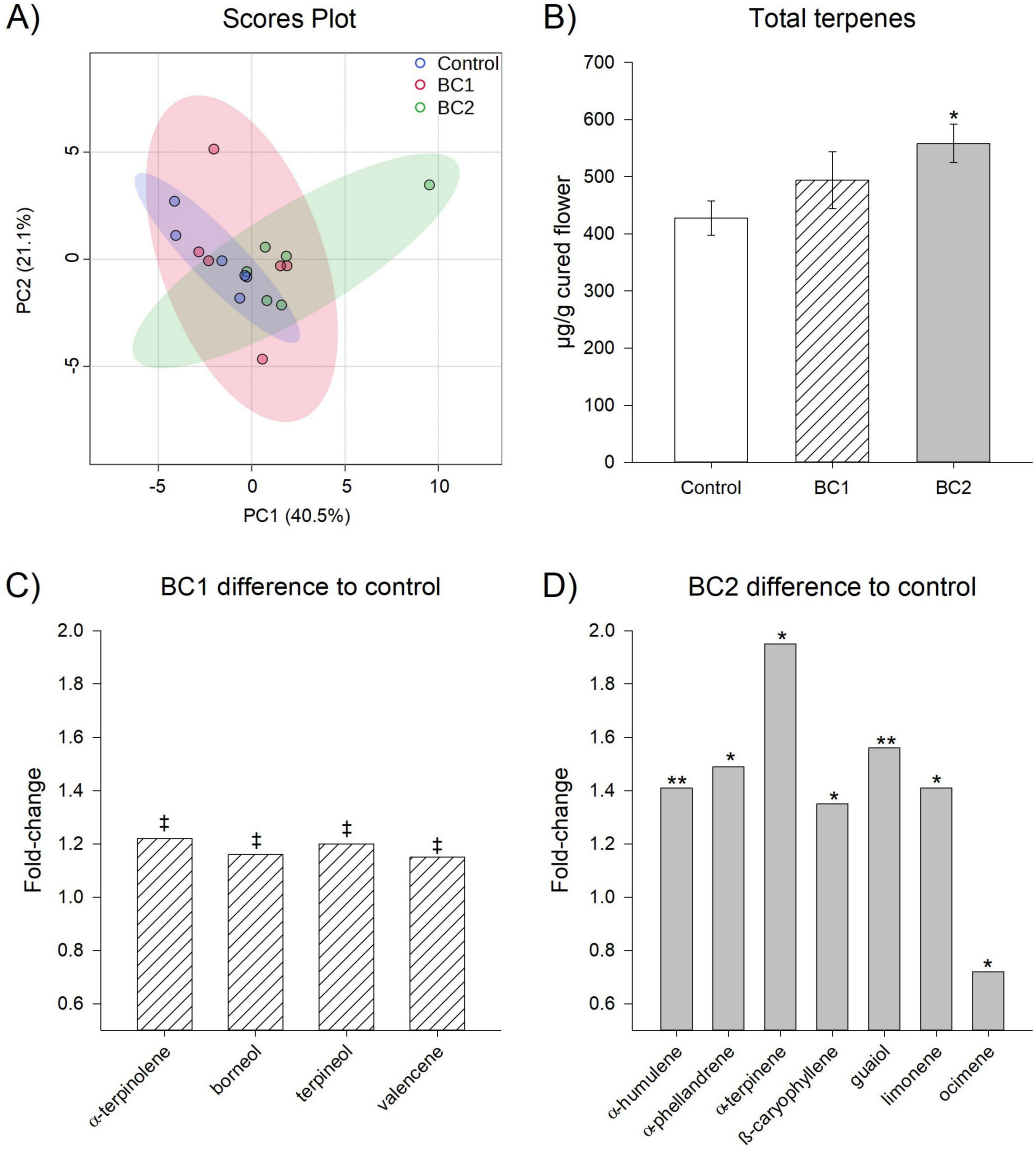
Impact of biostimulant complexes (BCs) to cannabis flower terpenes. **(A)** Principal component analysis (PCA) of terpene profiles from 6 flowers per treatment with shading indicating 95% confidence interval (CI) (blue: control, red: BC1, green: BC2). **(B)** mean total terpenes ± standard error from 6 flowers per treatment. Fold-change difference in average individual terpenes of interest as compared to control for **(C)** BC1, and **(D)** BC2. Statistical significance calculated by 2-sample *t*-test compared to control, and indicated by ** for p< 0.01, * for p< 0.05 and marginal significance indicated by ‡ for p< 0.1.

Terpenes are major constituents of cannabis flowers and extracted oils, and contribute therapeutic value either directly or via synergistic interactions with cannabinoids, referred to as the ‘entourage effect’ (Russo, 2011; Sommano et al., 2020). Profile analyses of the terpene data indicated that the BC1 and BC2 treatments appeared to impact flower terpenes differently. Principal component analysis (PCA) indicated that profiles were not distinct (as indicated by overlap of 95% CI, **Figure 3A**). Whilst pair-wise PERMANOVA results indicated a non-significant difference between the BC1 and control centroids (p = 0.602), a significant difference between the BC2 and control centroids was observed (p = 0.006). Furthermore, heatmap clustering indicated that BC1 and control profiles formed a cluster separate from BC2 (**Supplementary Figure S2**). To explore the underlying drivers of these profile-level shifts, individual terpene concentrations were compared between treatments (**Supplementary Table S10**). The BC1 treatment was associated with changes to four volatiles, including marginally significant increases to α-terpinolene (1.22-fold increase, p = 0.071), borneol (1.16-fold increase, p = 0.084), terpineol (1.20-fold increase, p = 0.098), and valencene (1.15-fold increase, p = 0.081). By contrast, BC2 resulted in significant changes to seven volatiles, including α-humulene (1.41-fold increase, p = 0.007), α-phellandrene (1.49-fold increase, p = 0.021), α-terpinene (1.95-fold increase, p = 0.024), β-caryophyllene (1.35-fold increase, p = 0.024), guaiol (1.56-fold increase, p = 0.003), limonene (1.41-fold increase, p = 0.038), and ocimene (0.72-fold reduction, p = 0.018). In addition to these changes to individual terpenes, both treatments were associated with on-average increases to total terpenes, with a statistically significant 1.30-fold increase for BC2 (p = 0.016), whereas the 1.16-fold increase for BC1 was not statistically significant (p = 0.280). These terpene changes provide preliminary evidence of treatment-associated effects and warrant further exploration in follow-on biostimulant studies using a larger number of plants.

Terpenes are involved in plant responses against both abiotic and biotic stressors, such as protection against UV radiation, and deterrence of insects, respectively (Brousseau et al., 2021; Kostanda and Khatib, 2022). Of note, two of the compounds increased by BC2 were the sesquiterpenes α-humulene and β-caryophyllene, which stand out as potentially related changes as they are both downstream products of the same enzyme, which is associated with the CsTPS9FN gene (Booth et al., 2017). Accordingly, a mode of effect of BC2 may be upregulation within the flowers of the CsTPS9FN gene and its associated sesquiterpenes. Furthermore, these sesquiterpenes are categorised as secondary metabolites, which are known to be involved in plant defence responses to insects and herbivory (Ashour et al., 2010). Both treatments herein were associated with increases to terpenes, suggesting potential induction of molecular defence responses. Previous reports have identified that defence responses in cannabis are induced by *A. vera* extract (Wise et al., 2024a) and molasses (Wise et al., 2024c). Both *A. vera* and molasses were included in the BC1 formulation, whereas BC2 contained *A. vera* but used GoS in place of molasses as the carbohydrate source. These findings are consistent with Wise and Selby-Pham (2026b), wherein GOS was found to impart a larger change than molasses to cannabis flower terpene profiles. Accordingly, the utilisation of GOS as a carbohydrate source within BCs (as a substitute for simpler sugars) appears to be an effective strategy to increasing cannabis volatile content.

Cannabis volatiles, including terpenes, contribute therapeutic effects (Cox-Georgian et al., 2019; Sommano et al., 2020), such that BC-associated modulations to the volatile profile would be anticipated to impact the cannabis flower medicinal utilities. The BC1 treatment increased a range of terpenes which are known to contribute to sedative and relaxant effects of cannabis, including: α-terpinolene (Oyibo, 2021), borneol (Xiao et al., 2022), and terpineol (Khaleel et al., 2018; Sousa et al., 2007). Promotion of relaxation is a key use of medicinal cannabis to enhance wellbeing and thereby quality of life. This includes: patients with mental health conditions (Walsh et al., 2017), geriatric patients experiencing chronic pain, sleep disturbance, cancer-related symptoms, or mood disorders (Minerbi et al., 2019), cancer patients (Smith et al., 2023), and patients with fibromyalgia (Fiz et al., 2011). Accordingly, the BC1-associated modulation of the cannabis terpene profile may have positively affected some of these relaxation effects from the flower terpenes.

Exploration of terpene changes within BC2 flowers indicated that the majority of the altered terpenes have demonstrated anti-inflammatory properties either *in vitro* or *in vivo* in human or animal models, including: α-humulene (Dalavaye et al., 2024; de Lacerda Leite et al., 2021), α-phellandrene (Thangaleela et al., 2022), α-terpinene (Mendoza M and Saavedra A, 2013), β-caryophyllene (Francomano et al., 2019; Gyrdymova and Rubtsova, 2022), guaiol (Akram et al., 2023), and limonene (d’Alessio et al., 2013; Santana et al., 2020). Although BC2 was mostly associated with increases to terpenes, there was a reduction to one terpene, ocimene, which also has demonstrated anti-inflammatory capacity (Laraib et al., 2025). Nevertheless, the changes to these terpenes from BC2 may be associated with positive changes to the anti-inflammatory capacity of the flowers. Noting that a common utilisation of medicinal cannabis is the treatment of inflammation (Anil et al., 2022), cultivation practises that promote this beneficial property (such as BC2, if validated) would be highly desirable to medicinal cannabis cultivators.

**Figure 4:**
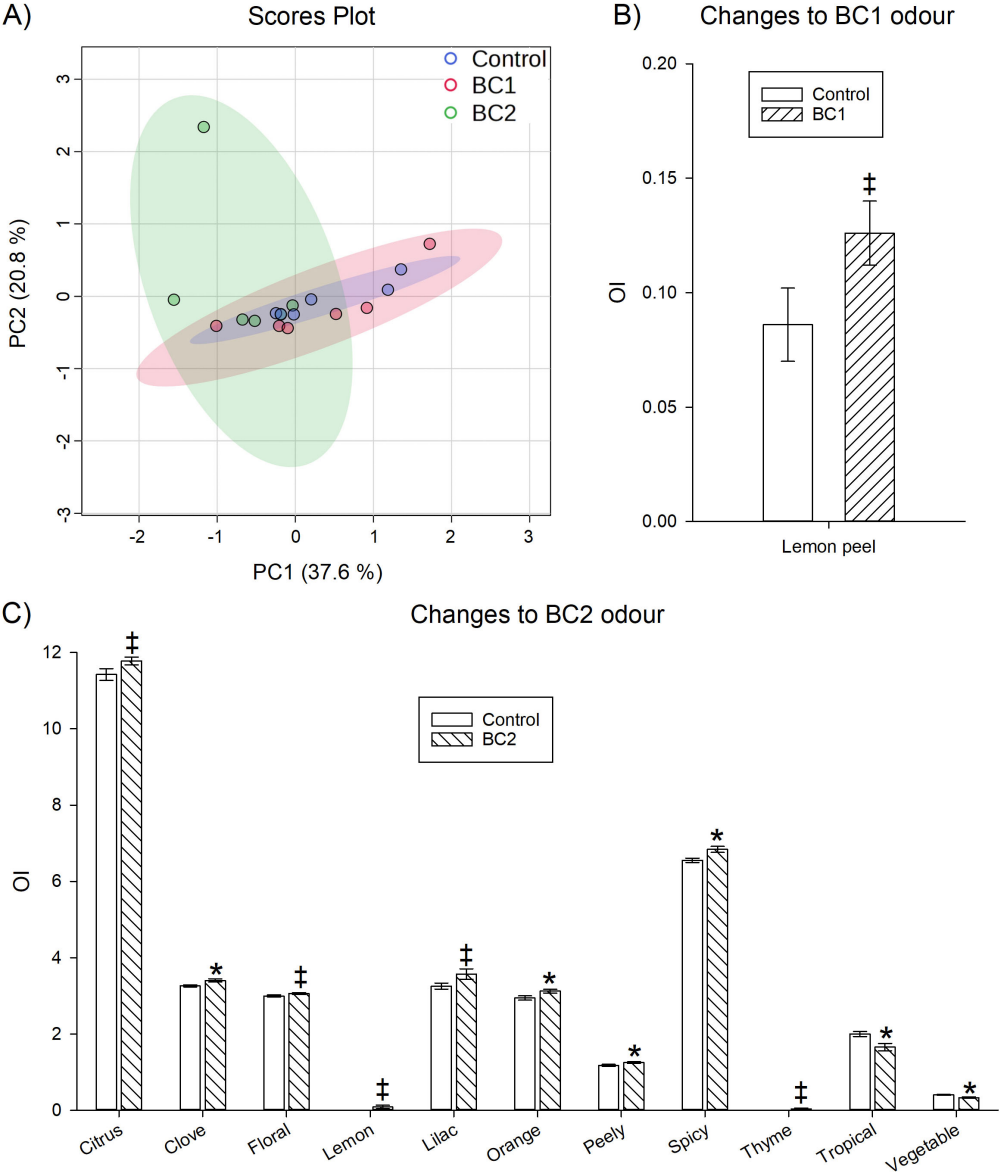
Impact of biostimulant complexes (BCs) to cannabis flower odour. **(A)** Principal component analysis (PCA) of predicted odour profile from 6 flowers per treatment with shading indicating 95% confidence interval (CI) (blue: control, red: BC1, green: BC2). Fold-change difference in average individual odours of interest as compared to control for **(B)** BC1, and **(C)** BC2. Statistical significance calculated by 2-sample *t*-test compared to control, and indicated by ** for p< 0.01, * for p< 0.05 and marginal significance indicated by ‡ for p< 0.1.

Terpene profiles were transformed into OI profiles to explore treatment-related effects to perceived odour (commonly referred to as ‘aromas’, herein referred to as ‘ODs’, and measured as ‘OI’). Profile analysis of the flower OI profiles indicated that the BC1 and BC2 treatments appeared to impact flower odour differently. Profile analysis by PCA indicated that the profiles were not distinct (as indicated by overlap of 95% CI, **Figure 4A**). However, pair-wise PERMANOVA indicated that BC2 (p = 0.015), but not BC1 (p = 0.873) was associated with a significant shift in profile centroids. Furthermore, heatmap clustering indicated that BC1 and control profiles grouped together, separately from BC2 (**Supplementary Figure S3**). Comparison of the 34 individual OIs scored for at least one treatment, indicated that BC1 flowers had a marginally significant change to 1 OI (**Figure 4B**; **Supplementary Table S11**), which was lemon peel (1.47-fold increase, p = 0.090). By comparison, BC2 flowers had significant changes to 6 OIs (**Figure 4C**; **Supplementary Table S11**): clove (1.04-fold increase, p = 0.020), orange (1.06-fold increase, p = 0.040), peely (1.06-fold increase, p = 0.042), spicy (1.04-fold increase, p = 0.017), tropical (0.83-fold decrease, p = 0.019), and vegetable (0.83-fold decrease, p = 0.018)., as well as marginally significant changes to 5 OIs: citrus (1.03-fold increase, p = 0.087), floral (1.02-fold increase, p = 0.072), lemon (detected in BC2 but not in control or BC1, p = 0.061), lilac (1.10-fold increase, p = 0.086), and thyme (detected in BC2 but not in control or BC1, p = 0.060).

The OI changes indicate shifts to the odour profiles of cannabis (Morello et al., 2022), which may be interpreted by customers as changes to quality and value (Gilbert and DiVerdi, 2018; Wise et al., 2023b). This is an important consideration for flower value, as customers have been shown to be highly sensitive to certain odours, which they associate with quality defects and therefore reduced flower quality (Koziel et al., 2022). Accordingly, possible implications of the BC-associated odour modulation to consumer impressions were explored by comparison to the datasets from 3 previous cannabis assessment studies; Cieslinski et al. (2023); Gilbert and DiVerdi (2018), and Wise et al. (2025b). Cieslinski et al. (2023) presents a novel cannabis-aroma wheel, which akin to wine aroma wheels, guides the categorisation of cannabis odours and proposes a lexicon for cannabis odour description. Furthermore, Cieslinski et al. (2023) explored how consumer perception of these odours impacts impression of the cannabis flower, quantified as ‘liking’. Similarly, Gilbert and DiVerdi (2018) and Wise et al. (2025b) present impacts of odour detection to consumer assessments of flowers. These 3 studies were utilised to guide possible interpretations to change in consumer impressions from BC-associated impacts to the cannabis flower odour profiles.

Odours are often utilised in cannabis strain name assignment, with fruity odours commonly driving strain names (de la Fuente et al., 2020), highlighting the practical and cultural significance of fruity aromas in cannabis. In the present study, BC2 was associated with a significant increase to orange and peely, as well as marginally significant increases to citrus and lemon, whilst BC1 was associated with a marginally significant increase to lemon peel only (**Figure 4B**; **Supplementary Table S11**). Noting that ‘peely’ and ‘lemon peel’ refer to citrus peels (Britten-Kelly, 2007), these odours would be represented by ‘citrus’, which is nested within ‘fruit’ odour category within the Cieslinski et al. (2023) cannabis odour wheel. Cieslinski et al. (2023) identified that the fruit odour category imparted the largest positive effect (16%) to consumer liking, and that citrus (16%) and lemon (18%) were 2 of the top 3 specific odours positively driving liking. These results are consistent with Gilbert and DiVerdi (2018), wherein detection of lemon (**Supplementary Table S12**) was associated with a 1.13-fold increase in interest (p< 0.001) and a 1.08-fold increase to price (p = 0.01), and detection of orange (**Supplementary Table S13**) was associated with a 1.17-fold increase in interest (p< 0.001) and a 1.16-fold increase to price (p< 0.001). Furthermore, the dataset from Wise et al. (2025b) indicates that detection of the citrus OD (**Supplementary Table S14**) was associated with a 2.93-fold increase to quality (p = 0.043), but no significant change to desirability (p = 0.204). Accordingly, the increase to multiple citrus odours by BC2, and BC1 exclusively increasing one citrus odour, may be associated with positively driving consumer impressions of the cannabis flower, with BC2 expected to drive a larger citrus-associated positive effect.

Cieslinski et al. (2023) also identified that tropical odour (nested within the fruit odour category) imparted a 17% increase to liking, the second highest specific odour effect they detected only after lemon (18%). This is consistent with Gilbert and DiVerdi (2018), wherein detection of ‘tropical fruit’ (**Supplementary Table S15**) was associated with a 1.20-fold increase in interest (p< 0.001) and a 1.13-fold increase in price (p = 0.003). Accordingly, the BC2-associated reduction to tropical may negatively drive consumer impressions, and potentially offset some of the positive impacts of increases to other fruit odours.

Impacts to consumer impressions from the BC2-assocaited increases to the ODs floral and lilac may be informed by Cieslinski et al. (2023) data for the floral odour, which they present as being associated with a 12% increase to liking. This is consistent with Gilbert and DiVerdi (2018), wherein detection of ‘flowery’ (**Supplementary Table S16**) was associated with a 1.08-fold increase in interest (p< 0.001) and a 1.06-fold increase in price (p = 0.019). Similarly, the dataset from Wise et al. (2025b) indicated that detection of the floral OD (**Supplementary Table S17**) was associated with a 5.75-fold increase to quality (p< 0.001), and a 2.46-fold increase to desirability (p = 0.008). Accordingly, the BC2 associated increases to these floral OIs may positively drive consumer impressions.

Impacts to consumer impressions from the BC2-assocaited increases to the ODs clove, spicy, and thyme, may be informed by Cieslinski et al. (2023) data for the odour category of ‘herbs and spices’. Their data indicated that the presence of odours from this category were not associated with significant impacts to consumer liking. This is consistent with Gilbert and DiVerdi (2018), wherein detection of spicy (**Supplementary Table S18**) was not associated with significant impacts to interest (p = 0.435) or price (p = 0.419), and Wise et al. (2025b), wherein both clove (**Supplementary Table S19**) and spicy (**Supplementary Table S20**) were not associated with significant impacts to quality or desirability. As such BC2-associated increases to these ‘herbs and spices’ ODs are not anticipated to impact consumer impressions.

Whilst not assessed directly, implications from BC2-assocaited reductions to the vegetable OI may be potentially informed from consumer-associations with related odours, presented in previous studies. Vegetable odour, often referred to as ‘vegetal’, is associated with green and rural-type descriptors (Fragrantica, n.d.). Cieslinski et al. (2023) identified that ‘agriculture’ captured ‘off’ odours and was associated with a 6% reduction in liking scores. These odours (also described as ‘hay’) are attributed to chlorophyll, and are utilised by consumers as indicators of incomplete/improper handling or drying, and thereby function as negative indicators of quality (Hyde and Goggins, 2025; Steven, 2024). This is consistent with Gilbert and DiVerdi (2018), wherein detection of ‘earthy’ (**Supplementary Table S21**) was associated with a 0.89-fold decrease in interest (p< 0.001) and a 0.92-fold decrease in price (p = 0.019). Similarly, the dataset from Wise et al. (2025b) indicated that whilst not statistically significant, on-average detection of the earthy OD (**Supplementary Table S22**) was associated with reductions to quality and desirability. Accordingly, to the extent that these related ODs inform the impacts of the vegetable OD, the BC2 decreases to vegetable may positively drive consumer impressions via reduction of this negatively-perceived odour.

In addition to the impacts from individual odour modulations to customer impressions of quality (explored above), data sourced from Wise et al. (2025b) was utilised to explore combined effects by statistical modelling to consumer impression of quality from modulation of citrus, clove, floral, and spicy. Ordinal logistic regression for desirability achieved a significant model (p = 0.044) and indicated that significant impacts to desirability (**Supplementary Table S23**) were exclusively driven by a positive association with floral (p = 0.010), while citrus, clove, and spicy were not significantly associated with desirability. Of note, the odds ratio of 4.30 for floral indicates that when the floral odour was detected, consumers were 4.30-fold more likely to assign the cannabis flower as desirable than when floral was not detected. Similarly, logistic regression for quality achieved a significant model (p< 0.001), and indicated that significant impacts to quality (**Supplementary Table S24**) were exclusively driven by a positive association with floral (p< 0.001), while citrus, clove, and spicy not significantly associated with quality. Accordingly, despite citrus positively driving quality when assessed in isolation, the effects of this OI change disappear when considered in conjunction with the effects of the floral odour, potentially explained by collinearity of detection of the citrus and floral ODs, or the ‘mixture suppression’/’perceptual dominance’ effects from the larger floral effect size (Cain and Drexler, 1974; Livermore and Laing, 1998). Of note, the odds ratio of 12.65 for floral indicated that when the floral odour was detected, consumers were 12.65-fold more likely to assign the cannabis flower as high quality than when floral was not detected. Accordingly, these results indicate that overall (**Supplementary Table S25**), BC1-driven increases to the OI ‘lemon peel’ citrus-type odour (in the absence of a change to floral odours) may impart positive-to-neutral impacts to consumer impressions, whilst the BC2-driven increases to citrus- and floral-type odours (potentially also supported by reductions to vegetable odour) may positively drive consumer impressions of cannabis flowers.

## 4 Conclusion

This study investigated the effects of two biostimulant complexes on cannabis flower production: BC1, which contained molasses, *A. vera* extract, and fish hydrolysate; and BC2, which contained GoS, *A. vera* extract, and triacontanol. While both treatments improved flower yield, BC2 provided broader benefits, including increased flower size, increased terpene content, and enhanced odour properties. A hypothesis to explain these effects is the potential induction of plant stress-responses from carbohydrate-associated stimulation of microbes within the rhizosphere. Given the greater performance of BC2, particularly in regard to yield quantity and quality, these findings highlight the utility of the BC2 components (GoS, *A. vera* extract, and triacontanol) as biostimulant fertigation supplements to enhance cannabis cultivation. Adoption of biostimulants such as these may offer cultivators with targeted strategies to increase yield value. Future validation should continue to investigate GoS-driven mechanisms through microbial analyses, stress marker assessment, and gene expression profiling, alongside direct sensory testing and evaluation across additional cultivars and cultivation conditions.

## Data Availability

The raw data supporting the conclusions of this article will be made available by the authors, without undue reservation.
