# Supplementary material for: Comparison of two carbohydrate-based biostimulant complexes for their ability to enhance Cannabis sativa flower yield and quality
Source: Front Plant Sci. 2026 Jun 5;17:1842299. doi: 10.3389/fpls.2026.1842299 (PMC13278894; doi:10.3389/fpls.2026.1842299)
Supplement: Supplementary file 1 [file DataSheet1.docx]

Table S1. Weekly fertigation strength.

| Week | EC | Coco A&B nutrients (mL/L) |
| --- | --- | --- |
| 1 | 1.1 | 1.75 |
| 2 | 1.3 | 2.25 |
| 3 | 1.5 | 2.75 |
| 4 | 1.5 | 2.75 |
| 5 | 1.8 | 3.50 |
| 6 | 1.8 | 3.50 |
| 7 | 2.0 | 4.00 |
| 8 | 2.0 | 4.00 |
| 9 | 2.2 | 4.25 |
| 10 | 2.0 | 4.00 |
| 11 | 1.8 | 3.50 |
| 12 | 1.5 | 2.75 |

Table S2. Weekly fertigation pump schedule

| Week | Pump duration (s) |
| --- | --- |
| 1 | 10 |
| 2–3 | 15 |
| 4–5 | 20 |
| 6 | 30 |
| 7–12 | 40 |

Table S3. Chromatic pixel colour assignment criteria using Lab*.

|  |  | Red | Orange | Yellow | Green | Cyan | Blue | Purple | Pink | Brown |
| --- | --- | --- | --- | --- | --- | --- | --- | --- | --- | --- |
| Band | L* | Hue range | Hue range | Hue range | Hue range | Hue range | Hue range | Hue range | Hue range | Hue range |
| 1 | L* < 20 | 15° – 35° | 50° – 65° | 65° – 100° | 88° – 195° | 180° – 240° | 240° – 290° | 290° – 345° | 345° – 15° | 35° – 50° |
| 2 | 20 ≤ L* < 35 | 15° – 35° | 55° – 75° | 75° – 90° | 88° – 195° | 180° – 240° | 240° – 290° | 290° – 345° | 345° – 15° | 35° – 55° |
| 3 | 35 ≤ L* < 50 | 15° – 40° | 40° – 70° | 70° – 95° | 88° – 195° | 180° – 240° | 240° – 290° | 290° – 345° | 345° – 15° | ND^a^ |
| 4 | 50 ≤ L* < 65 | 15° – 48° | 45° – 75° | 75° – 100° | 100° – 180° | 180° – 240° | 240° – 290° | 290° – 345° | 345° – 15° | ND^a^ |
| 5 | 65 ≤ L* < 80 | 15° – 48° | 45° – 75° | 75° – 102° | 102° – 180° | 180° – 240° | 240° – 290° | 290° – 345° | 345° – 15° | ND^a^ |
| 6 | L* ≥ 80 | 15° – 47° | 45° – 75° | 75° – 105° | 105° – 180° | 180° – 240° | 240° – 290° | 290° – 345° | 345° – 15° | ND^a^ |

^a^Not defined

Table S4. Biomass and yield

|  | Control | | | BC1 | | | | | BC2 | | | | |
| --- | --- | --- | --- | --- | --- | --- | --- | --- | --- | --- | --- | --- | --- |
| Measure | N | Mean | StDev | N | Mean | StDev | P-value^a^ | F-C^b^ | N | Mean | StDev | P-value^a^ | F-C^b^ |
| Stem thickness (mm) | 6 | 13.38 | 1.13 | 6 | 12.73 | 1.08 | 0.332 | 0.95 | 6 | 14.09 | 1.55 | 0.386 | 1.05 |
| Biomass (g FW) | 6 | 605.00 | 43.20 | 6 | 583.30 | 67.20 | 0.521 | 0.96 | 6 | 615.80 | 65.60 | 0.743 | 1.02 |
| Weight (g CW) | 6 | 12.93 | 2.22 | 6 | 15.13 | 1.93 | 0.097 | 1.17 | 6 | 28.73 | 9.41 | 0.003 | 2.22 |

^a^2-sample t-test compared to control, ^b^Fold-change compared to control

Table S5. Flower dimensions

|  | Control | | | BC1 | | | | | BC2 | | | | |
| --- | --- | --- | --- | --- | --- | --- | --- | --- | --- | --- | --- | --- | --- |
| Measure | N | Mean | StDev | N | Mean | StDev | P-value | F-C^a^ | N | Mean | StDev | P-value | F-C^a^ |
| Flower size (mm) | 18 | 19.00 | 6.60 | 18 | 19.50 | 5.85 | 0.642^b^ | 1.03 | 18 | 24.33 | 7.80 | 0.003^b^ | 1.28 |
| Flower area (cm^2^) | 6 | 2.97 | 0.32 | 6 | 3.10 | 0.30 | 0.487^c^ | 1.04 | 6 | 3.69 | 0.84 | 0.098^c^ | 1.24 |

^a^Fold-change compared to control, ^b^treatment effects compared to control by general linear model GLM), accounting for ‘plant’ as a nested random factor, ^c^2-sample t-test compared to control

Table S6. Flower size grading

|  | Control | | | BC1 | | | | | BC2 | | | | |
| --- | --- | --- | --- | --- | --- | --- | --- | --- | --- | --- | --- | --- | --- |
| Measure | N | Count | % | N | Count | % | P-value^a^ | F-C^b^ | N | Count | % | P-value^a^ | F-C^b^ |
| Grade A | 18 | 11 | 61.11 | 18 | 12 | 66.67 | 0.728 | 1.09 | 18 | 16 | 88.89 | 0.042 | 1.45 |
| Grade B | 18 | 5 | 27.78 | 18 | 5 | 27.78 | 1.000 | 1.00 | 18 | 2 | 11.11 | 0.196 | 0.40 |
| Shake | 18 | 2 | 11.11 | 18 | 1 | 5.56 | 0.544 | 0.50 | 18 | 0 | 0.00 | 0.134 | 0.00 |

^a^2-proportions compared to control, ^b^Fold-change compared to control


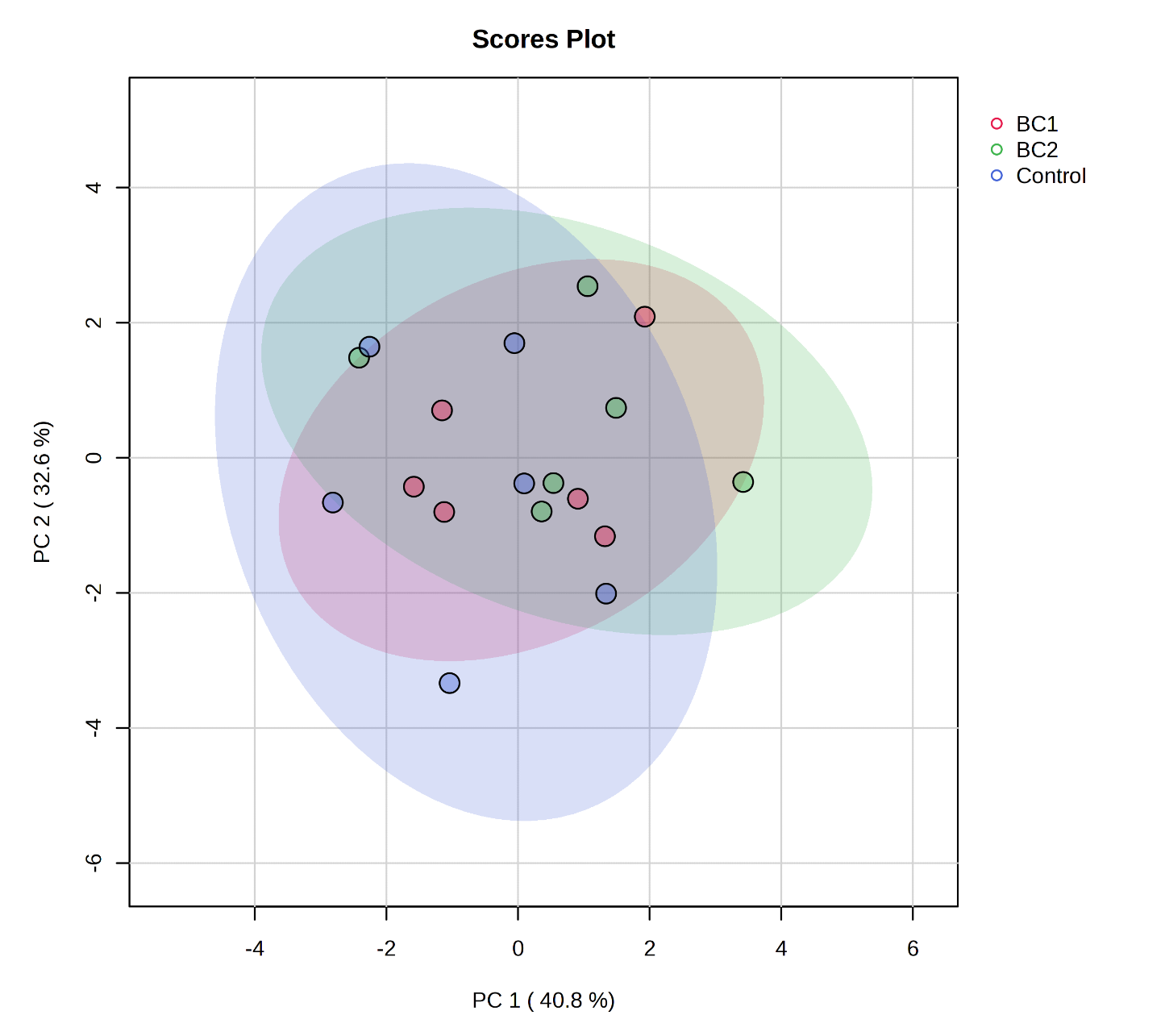


Figure S1. Flower colour PCA

Table S7. Flower colour

|  | Control | | | BC1 | | | | | BC2 | | | | |
| --- | --- | --- | --- | --- | --- | --- | --- | --- | --- | --- | --- | --- | --- |
| Measure^a^ | N | Mean | StDev | N | Mean | StDev | P-value^b^ | F-C^c^ | N | Mean | StDev | P-value^b^ | F-C^c^ |
| Black (%) | 6 | 0.92 | 0.28 | 6 | 1.12 | 0.61 | 0.505 | 1.21 | 6 | 1.22 | 0.42 | 0.189 | 1.32 |
| Green (%) | 6 | 83.86 | 1.67 | 6 | 84.07 | 1.54 | 0.828 | 1.00 | 6 | 84.95 | 2.28 | 0.372 | 1.01 |
| Grey (%) | 6 | 2.48 | 0.80 | 6 | 2.04 | 0.58 | 0.302 | 0.82 | 6 | 1.32 | 0.37 | 0.014 | 0.53 |
| Orange (%) | 6 | 0.45 | 0.11 | 6 | 0.36 | 0.09 | 0.158 | 0.80 | 6 | 0.35 | 0.07 | 0.085 | 0.76 |
| Red (%) | 6 | 0.38 | 0.08 | 6 | 0.31 | 0.05 | 0.129 | 0.83 | 6 | 0.31 | 0.06 | 0.126 | 0.82 |
| White (%) | 6 | 2.94 | 1.11 | 6 | 2.51 | 0.56 | 0.422 | 0.85 | 6 | 2.90 | 0.48 | 0.938 | 0.99 |
| Yellow (%) | 6 | 8.96 | 1.76 | 6 | 9.58 | 1.00 | 0.475 | 1.07 | 6 | 8.95 | 1.98 | 0.996 | 1.00 |

^a^Blue not detected, Pink and Brown accounted for a total of 0.002-0.007%,  ^b^2-sample *t*-test compared to control, ^c^Fold-change compared to control

Table S8. Abundance of functional groups within flowers determined from N-IR

|  |  | Control | | | BC1 | | | | | BC2 | | | | |
| --- | --- | --- | --- | --- | --- | --- | --- | --- | --- | --- | --- | --- | --- | --- |
| Measure | µm range | N | Mean^a^ | StDev | N | Mean^a^ | StDev | P-value^b^ | F-C^c^ | N | Mean^a^ | StDev | P-value^b^ | F-C^c^ |
| RNH_2_ | 1.017–1.043 | 6 | 0.6621 | 0.1284 | 6 | 0.8632 | 0.1307 | 0.023 | 1.30 | 6 | 0.8438 | 0.1102 | 0.025 | 1.27 |
| ArCH | 1.094–1.099 | 6 | 0.0052 | 0.0028 | 6 | 0.0050 | 0.0044 | 0.927 | 0.96 | 6 | 0.0108 | 0.0030 | 0.007 | 2.08 |
| CH_3_ | 1.118–1.194 | 6 | 4.7150 | 0.2910 | 6 | 5.2360 | 0.3400 | 0.017 | 1.11 | 6 | 5.2580 | 0.3600 | 0.017 | 1.12 |

^a^Per-treatment average determined from the sum of absorbances across the µm range, ^b^2-sample t-test compared to control, ^c^Fold-change compared to control

Table S9. Flower cannabinoids determined from N-IR

|  | Control | | | BC1 | | | | | BC2 | | | | |
| --- | --- | --- | --- | --- | --- | --- | --- | --- | --- | --- | --- | --- | --- |
| Measure | N | Mean | StDev | N | Mean | StDev | P-value^a^ | F-C^b^ | N | Mean | StDev | P-value^a^ | F-C^b^ |
| THC | 6 | ND^c^ | N/A | 6 | ND^c^ | N/A | N/A | N/A | 6 | ND^c^ | N/A | N/A | N/A |
| CBD (% g CW) | 6 | 2.870 | 0.324 | 6 | 3.831 | 0.445 | 0.002 | 1.33 | 6 | 2.978 | 0.453 | 0.642 | 1.04 |

^a^2-sample t-test compared to control, ^b^Fold-change compared to control^, c^Not detected


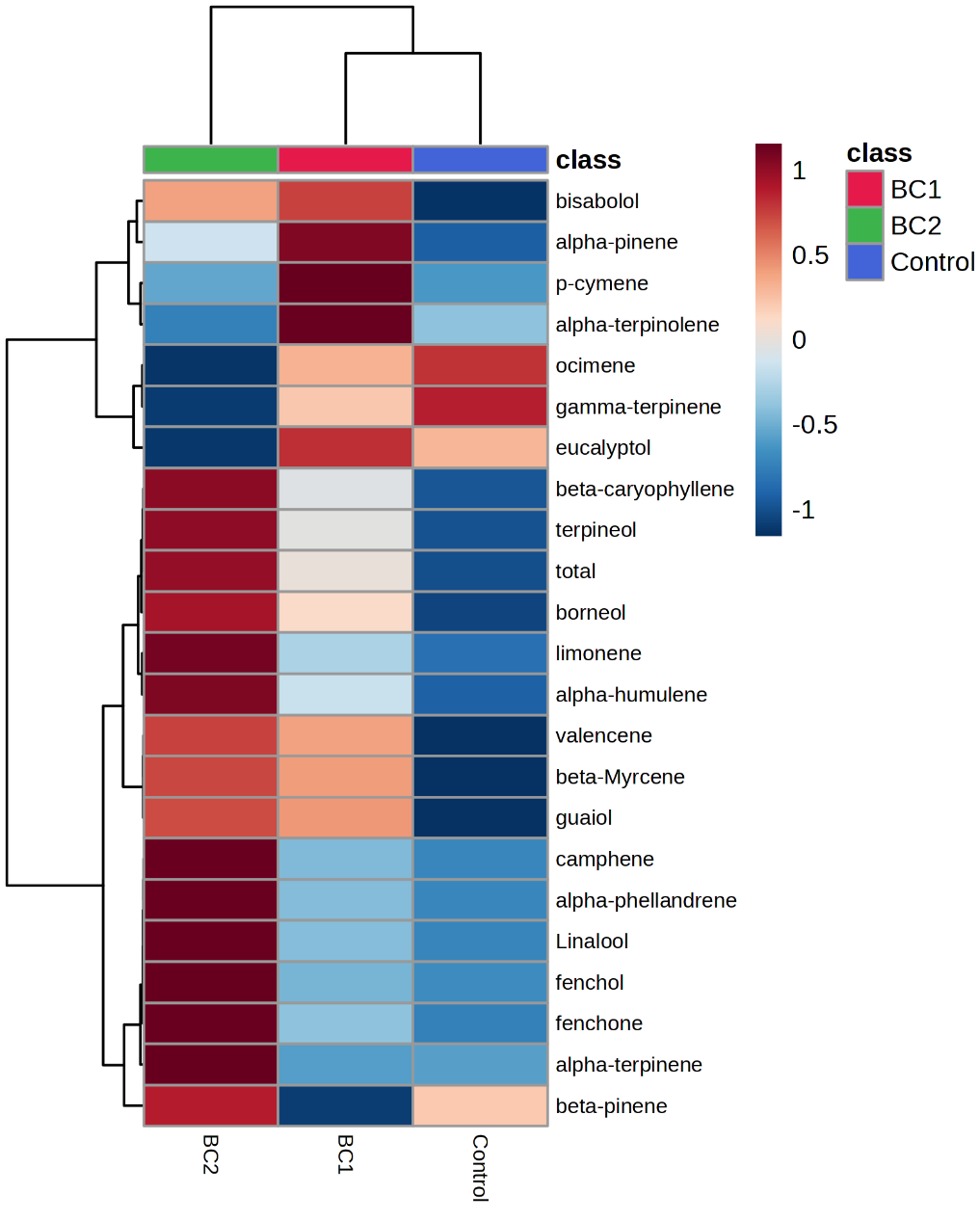


Figure S2. Heatmap of impact of BCs to volatile profiles

Table S10. Impact of BCs to individual volatiles

|  | Control | | | BC1 | | | | | BC2 | | | | |
| --- | --- | --- | --- | --- | --- | --- | --- | --- | --- | --- | --- | --- | --- |
| Measure | N | Mean | StDev | N | Mean | StDev | P-value^a^ | F-C^b^ | N | Mean | StDev | P-value^a^ | F-C^b^ |
| α-humulene | 6 | 28.77 | 3.60 | 6 | 33.33 | 6.42 | 0.160 | 1.16 | 6 | 40.69 | 7.76 | 0.007 | 1.41 |
| α -phellandrene | 6 | 3.32 | 0.55 | 6 | 3.57 | 0.62 | 0.477 | 1.07 | 6 | 4.96 | 1.36 | 0.021 | 1.49 |
| α -pinene | 6 | 11.03 | 2.32 | 6 | 11.57 | 3.15 | 0.742 | 1.05 | 6 | 11.24 | 2.44 | 0.882 | 1.02 |
| α -terpinene | 6 | 0.96 | 0.18 | 6 | 0.96 | 0.32 | 1.000 | 1.00 | 6 | 1.87 | 0.82 | 0.024 | 1.95 |
| α -terpinolene | 6 | 0.31 | 0.06 | 6 | 0.38 | 0.06 | 0.071 | 1.22 | 6 | 0.30 | 0.06 | 0.779 | 0.95 |
| β-caryophyllene | 6 | 102.56 | 12.97 | 6 | 118.89 | 21.67 | 0.144 | 1.16 | 6 | 138.00 | 29.90 | 0.024 | 1.35 |
| β-myrcene | 6 | 123.04 | 23.03 | 6 | 142.60 | 66.10 | 0.509 | 1.16 | 6 | 145.30 | 76.50 | 0.510 | 1.18 |
| β-pinene | 6 | 7.77 | 2.41 | 6 | 6.18 | 3.98 | 0.422 | 0.80 | 6 | 8.58 | 3.03 | 0.619 | 1.10 |
| bisabolol | 6 | 0.71 | 0.35 | 6 | 0.98 | 0.14 | 0.110 | 1.39 | 6 | 0.94 | 0.17 | 0.178 | 1.32 |
| borneol | 6 | 9.05 | 1.30 | 6 | 10.48 | 1.28 | 0.084 | 1.16 | 6 | 11.48 | 3.09 | 0.106 | 1.27 |
| camphene | 6 | 3.68 | 1.00 | 6 | 3.62 | 1.93 | 0.947 | 0.98 | 6 | 3.74 | 1.17 | 0.926 | 1.02 |
| eucalyptol | 6 | 0.76 | 0.22 | 6 | 0.81 | 0.21 | 0.696 | 1.06 | 6 | 0.63 | 0.17 | 0.279 | 0.83 |
| fenchol | 6 | 19.69 | 5.04 | 6 | 20.35 | 3.32 | 0.794 | 1.03 | 6 | 25.16 | 10.91 | 0.291 | 1.28 |
| fenchone | 6 | 6.67 | 1.30 | 6 | 6.88 | 1.26 | 0.782 | 1.03 | 6 | 7.83 | 1.83 | 0.234 | 1.17 |
| γ-terpinene | 6 | 0.61 | 0.05 | 6 | 0.60 | 0.06 | 0.760 | 0.98 | 6 | 0.58 | 0.03 | 0.236 | 0.95 |
| guaiol | 6 | 29.77 | 6.55 | 6 | 44.03 | 19.08 | 0.114 | 1.48 | 6 | 46.56 | 8.47 | 0.003 | 1.56 |
| limonene | 6 | 64.88 | 17.37 | 6 | 72.60 | 31.80 | 0.613 | 1.12 | 6 | 91.70 | 21.39 | 0.038 | 1.41 |
| linalool | 6 | 1.63 | 0.17 | 6 | 1.65 | 0.47 | 0.924 | 1.01 | 6 | 1.77 | 0.12 | 0.130 | 1.09 |
| ocimene | 6 | 0.33 | 0.05 | 6 | 0.31 | 0.05 | 0.504 | 0.93 | 6 | 0.24 | 0.06 | 0.018 | 0.72 |
| p-cymene | 6 | 0.32 | 0.04 | 6 | 0.35 | 0.06 | 0.332 | 1.08 | 6 | 0.32 | 0.03 | 1.000 | 1.00 |
| terpineol | 6 | 8.93 | 1.59 | 6 | 10.75 | 1.85 | 0.098 | 1.20 | 6 | 12.73 | 5.32 | 0.125 | 1.43 |
| valencene | 6 | 2.98 | 0.39 | 6 | 3.44 | 0.43 | 0.081 | 1.15 | 6 | 3.55 | 0.78 | 0.140 | 1.19 |
| Total | 6 | 427.80 | 73.70 | 6 | 494.30 | 122.00 | 0.280 | 1.16 | 6 | 558.20 | 82.70 | 0.016 | 1.30 |

^a^2-sample *t*-test compared to control, ^b^Fold-change compared to control


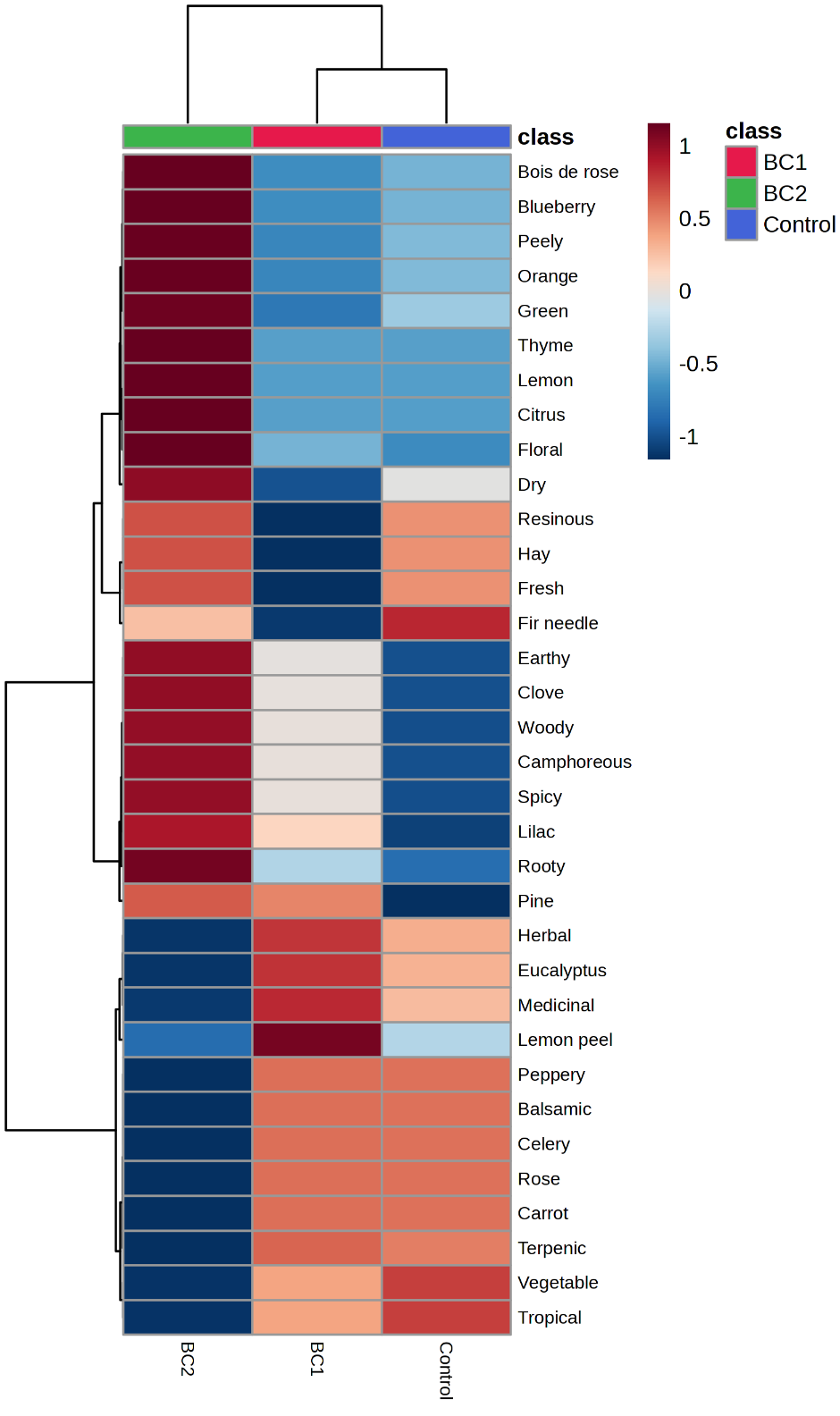


Figure S3. Heatmap of impact of BCs to odour profiles

Table S11. Impact of BCs to flower odour

|  | Control | | | BC1 | | | | | BC2 | | | | |
| --- | --- | --- | --- | --- | --- | --- | --- | --- | --- | --- | --- | --- | --- |
| Measure | N | Mean | StDev | N | Mean | StDev | P-value^a^ | F-C^b^ | N | Mean | StDev | P-value^a^ | F-C^b^ |
| Balsamic | 6 | 1.625 | 0.037 | 6 | 1.630 | 0.127 | 0.930 | 1.00 | 6 | 1.412 | 0.693 | 0.486 | 0.87 |
| Blueberry | 6 | 0.940 | 0.017 | 6 | 0.939 | 0.041 | 0.958 | 1.00 | 6 | 0.955 | 0.012 | 0.115 | 1.02 |
| Bois de rose | 6 | 1.880 | 0.035 | 6 | 1.877 | 0.081 | 0.936 | 1.00 | 6 | 1.909 | 0.023 | 0.128 | 1.02 |
| Camphoreous | 6 | 4.537 | 0.456 | 6 | 4.678 | 0.354 | 0.564 | 1.03 | 6 | 4.830 | 0.436 | 0.285 | 1.06 |
| Carrot | 6 | 1.015 | 0.023 | 6 | 1.019 | 0.080 | 0.911 | 1.00 | 6 | 0.882 | 0.433 | 0.486 | 0.87 |
| Celery | 6 | 1.160 | 0.026 | 6 | 1.165 | 0.091 | 0.902 | 1.00 | 6 | 1.009 | 0.495 | 0.489 | 0.87 |
| Citrus | 6 | 11.422 | 0.372 | 6 | 11.436 | 0.807 | 0.970 | 1.00 | 6 | 11.778 | 0.247 | 0.087 | 1.03 |
| Clove | 6 | 3.260 | 0.063 | 6 | 3.331 | 0.089 | 0.145 | 1.02 | 6 | 3.403 | 0.103 | 0.020 | 1.04 |
| Dry | 6 | 1.987 | 0.279 | 6 | 1.874 | 0.354 | 0.554 | 0.94 | 6 | 2.122 | 0.232 | 0.386 | 1.07 |
| Earthy | 6 | 1.162 | 0.112 | 6 | 1.204 | 0.097 | 0.505 | 1.04 | 6 | 1.248 | 0.079 | 0.163 | 1.07 |
| Eucalyptus | 6 | 4.486 | 0.254 | 6 | 4.548 | 0.248 | 0.679 | 1.01 | 6 | 4.305 | 0.264 | 0.257 | 0.96 |
| Fir needle | 6 | 0.206 | 0.097 | 6 | 0.223 | 0.122 | 0.795 | 1.08 | 6 | 0.207 | 0.114 | 0.987 | 1.01 |
| Floral | 6 | 2.994 | 0.061 | 6 | 3.002 | 0.125 | 0.892 | 1.00 | 6 | 3.059 | 0.049 | 0.072 | 1.02 |
| Fresh | 6 | 0.358 | 0.109 | 6 | 0.282 | 0.172 | 0.387 | 0.79 | 6 | 0.388 | 0.119 | 0.660 | 1.08 |
| Green | 6 | 1.439 | 0.035 | 6 | 1.430 | 0.073 | 0.793 | 0.99 | 6 | 1.470 | 0.025 | 0.111 | 1.02 |
| Hay | 6 | 0.215 | 0.065 | 6 | 0.169 | 0.103 | 0.382 | 0.79 | 6 | 0.233 | 0.071 | 0.658 | 1.08 |
| Herbal | 6 | 11.971 | 0.380 | 6 | 12.084 | 0.728 | 0.746 | 1.01 | 6 | 11.662 | 1.044 | 0.521 | 0.97 |
| Lemon | 6 | 0.000 | 0.000 | 6 | 0.000 | 0.000 | N/A | N/A | 6 | 0.084 | 0.111 | 0.061^c^ | N/A |
| Lemon peel | 6 | 0.086 | 0.038 | 6 | 0.126 | 0.035 | 0.090 | 1.47 | 6 | 0.075 | 0.042 | 0.646 | 0.87 |
| Lilac | 6 | 3.254 | 0.193 | 6 | 3.441 | 0.180 | 0.117 | 1.06 | 6 | 3.569 | 0.335 | 0.086 | 1.10 |
| Medicinal | 6 | 1.496 | 0.085 | 6 | 1.516 | 0.083 | 0.690 | 1.01 | 6 | 1.446 | 0.082 | 0.327 | 0.97 |
| Orange | 6 | 2.942 | 0.142 | 6 | 2.927 | 0.394 | 0.933 | 1.00 | 6 | 3.120 | 0.114 | 0.040 | 1.06 |
| Peely | 6 | 1.177 | 0.057 | 6 | 1.171 | 0.158 | 0.933 | 1.00 | 6 | 1.248 | 0.046 | 0.042 | 1.06 |
| Peppery | 6 | 2.031 | 0.046 | 6 | 2.038 | 0.159 | 0.922 | 1.00 | 6 | 1.765 | 0.866 | 0.486 | 0.87 |
| Pine | 6 | 5.621 | 0.498 | 6 | 6.059 | 0.339 | 0.113 | 1.08 | 6 | 6.105 | 0.445 | 0.110 | 1.09 |
| Resinous | 6 | 0.154 | 0.047 | 6 | 0.121 | 0.074 | 0.383 | 0.79 | 6 | 0.166 | 0.051 | 0.682 | 1.08 |
| Rooty | 6 | 0.272 | 0.047 | 6 | 0.280 | 0.028 | 0.729 | 1.03 | 6 | 0.308 | 0.059 | 0.272 | 1.13 |
| Rose | 6 | 1.354 | 0.031 | 6 | 1.359 | 0.106 | 0.916 | 1.00 | 6 | 1.177 | 0.577 | 0.487 | 0.87 |
| Spicy | 6 | 6.552 | 0.140 | 6 | 6.696 | 0.190 | 0.169 | 1.02 | 6 | 6.838 | 0.193 | 0.017 | 1.04 |
| Terpenic | 6 | 10.151 | 0.293 | 6 | 10.227 | 0.704 | 0.815 | 1.01 | 6 | 9.410 | 2.980 | 0.571 | 0.93 |
| Thyme | 6 | 0.000 | 0.000 | 6 | 0.000 | 0.000 | N/A | N/A | 6 | 0.036 | 0.047 | 0.060^c^ | N/A |
| Tropical | 6 | 1.998 | 0.162 | 6 | 1.926 | 0.165 | 0.465 | 0.96 | 6 | 1.656 | 0.237 | 0.019 | 0.83 |
| Vegetable | 6 | 0.400 | 0.032 | 6 | 0.385 | 0.033 | 0.445 | 0.96 | 6 | 0.331 | 0.047 | 0.018 | 0.83 |
| Woody | 6 | 10.587 | 0.644 | 6 | 10.843 | 0.793 | 0.555 | 1.02 | 6 | 11.087 | 0.586 | 0.193 | 1.05 |

^a^2-sample t-test compared to control, ^b^Fold-change compared to control, ^c^For odours not detected in treatment but not control samples the P-value was determined by 1-sample *t*-test

Table S12: Impact of lemon odour detection to consumer impressions of cannabis.

| Variable | Lemon^a^ | N | Mean | StDev | FC^b^ | 2-sample t |
| --- | --- | --- | --- | --- | --- | --- |
| Interest | 0 | 681 | 7.3084 | 2.2226 |  |  |
|  | 1 | 112 | 8.25 | 1.504 | 1.13 | <0.001 |
| Price | 0 | 681 | 11.941 | 3.922 |  |  |
|  | 1 | 112 | 12.929 | 3.69 | 1.08 | 0.010 |

^a^Detection of lemon odour No (0) or Yes (1), ^b^Fold-change, mean for odour detected divided by mean for odour not detected. Data sourced from Gilbert and DiVerdi (2018).

Table S13: Impact of orange odour detection to consumer impressions of cannabis.

| Variable | Orange^a^ | N | Mean | StDev | FC^b^ | 2-sample t |
| --- | --- | --- | --- | --- | --- | --- |
| Interest | 0 | 722 | 7.331 | 2.1855 |  |  |
|  | 1 | 71 | 8.563 | 1.481 | 1.17 | < 0.001 |
| Price | 0 | 722 | 11.914 | 3.879 |  |  |
|  | 1 | 71 | 13.775 | 3.773 | 1.16 | < 0.001 |

^a^Detection of orange odour No (0) or Yes (1), ^b^Fold-change, mean for odour detected divided by mean for odour not detected. Data sourced from Gilbert and DiVerdi (2018).

Table S14: Impact of citrus odour detection to consumer impressions of cannabis.

| Variable | citrus^a^ | N^b^ | Total^c^ | Proportion | 2-proportions test | FC^d^ |
| --- | --- | --- | --- | --- | --- | --- |
| High quality | 0 | 77 | 15 | 0.19 | 0.043 | 2.93 |
|  | 1 | 7 | 4 | 0.57 |  |  |
| Desirable | 0 | 77 | 23 | 0.30 | 0.204 | 1.91 |
|  | 1 | 7 | 4 | 0.57 |  |  |

^a^Detection of citrus odour No (0) or Yes (1), ^b^Number of samples per odour detection category, ^c^Total samples assigned as high quality (quality scored ≥ 1) or desirable (desirability scored ≥ 1). ^d^Fold-change, proportion for odour detected divided by proportion for odour not detected. Data sourced from Wise et al. (2025).

Table S15: Impact of tropical fruit odour detection to consumer impressions of cannabis.

| Variable | Tropical fruit^a^ | N | Mean | StDev | FC^b^ | 2-sample t |
| --- | --- | --- | --- | --- | --- | --- |
| Interest | 0 | 713 | 7.2917 | 2.1805 |  |  |
|  | 1 | 80 | 8.775 | 1.387 | 1.20 | 0.000 |
| Price | 0 | 713 | 11.927 | 3.833 |  |  |
|  | 1 | 80 | 13.45 | 4.269 | 1.13 | 0.003 |

^a^Detection of tropical fruit odour No (0) or Yes (1), ^b^Fold-change, mean for odour detected divided by mean for odour not detected. Data sourced from Gilbert and DiVerdi (2018).

Table S16: Impact of flowery odour detection to consumer impressions of cannabis.

| Variable | Flowery^a^ | N | Mean | StDev | FC^b^ | 2-sample t |
| --- | --- | --- | --- | --- | --- | --- |
| Interest | 0 | 573 | 7.281 | 2.2171 |  |  |
|  | 1 | 220 | 7.859 | 1.948 | 1.08 | 0.000 |
| Price | 0 | 573 | 11.874 | 3.845 |  |  |
|  | 1 | 220 | 12.618 | 4.011 | 1.06 | 0.019 |

^a^Detection of flowery odour No (0) or Yes (1), ^b^Fold-change, mean for odour detected divided by mean for odour not detected. Data sourced from Gilbert and DiVerdi (2018).

Table S17: Impact of floral odour detection to consumer impressions of cannabis.

| Variable | floral^a^ | N^b^ | Total^c^ | Proportion | 2-proportions test | FC^d^ |
| --- | --- | --- | --- | --- | --- | --- |
| High quality | 0 | 61 | 6 | 0.10 | 0.000 | 5.75 |
|  | 1 | 23 | 13 | 0.57 |  |  |
| Desirable | 0 | 61 | 14 | 0.23 | 0.008 | 2.46 |
|  | 1 | 23 | 13 | 0.57 |  |  |

^a^Detection of floral odour No (0) or Yes (1), ^b^Number of samples per odour detection category, ^c^Total samples assigned as high quality (quality scored ≥ 1) or desirable (desirability scored ≥ 1). ^d^Fold-change, proportion for odour detected divided by proportion for odour not detected. Data sourced from Wise et al. (2025).

Table S18: Impact of spicy odour detection to consumer impressions of cannabis.

| Variable | Spicy^a^ | N | Mean | StDev | FC^b^ | 2-sample t |
| --- | --- | --- | --- | --- | --- | --- |
| Interest | 0 | 704 | 7.4645 | 2.1332 |  |  |
|  | 1 | 89 | 7.258 | 2.367 | 0.97 | 0.435 |
| Price | 0 | 704 | 12.125 | 3.84 |  |  |
|  | 1 | 89 | 11.73 | 4.382 | 0.97 | 0.419 |

^a^Detection of spicy odour No (0) or Yes (1), ^b^Fold-change, mean for odour detected divided by mean for odour not detected. Data sourced from Gilbert and DiVerdi (2018).

Table S19: Impact of clove odour detection to consumer impressions of cannabis.

| Variable | clove^a^ | N^b^ | Total^c^ | Proportion | 2-proportions test | FC^d^ |
| --- | --- | --- | --- | --- | --- | --- |
| High quality | 0 | 78 | 17 | 0.22 | 0.614 | 1.53 |
|  | 1 | 6 | 2 | 0.33 |  |  |
| Desirable | 0 | 78 | 24 | 0.31 | 0.381 | 1.63 |
|  | 1 | 6 | 3 | 0.50 |  |  |

^a^Detection of clove odour No (0) or Yes (1), ^b^Number of samples per odour detection category, ^c^Total samples assigned as high quality (quality scored ≥ 1) or desirable (desirability scored ≥ 1). ^d^Fold-change, proportion for odour detected divided by proportion for odour not detected. Data sourced from Wise et al. (2025).

Table S20: Impact of spicy odour detection to consumer impressions of cannabis.

| Variable | spicy^a^ | N^b^ | Total^c^ | Proportion | 2-proportions test | FC^d^ |
| --- | --- | --- | --- | --- | --- | --- |
| High quality | 0 | 72 | 17 | 0.24 | 0.726 | 0.71 |
|  | 1 | 12 | 2 | 0.17 |  |  |
| Desirable | 0 | 72 | 24 | 0.33 | 0.743 | 0.75 |
|  | 1 | 12 | 3 | 0.25 |  |  |

^a^Detection of spicy odour No (0) or Yes (1), ^b^Number of samples per odour detection category, ^c^Total samples assigned as high quality (quality scored ≥ 1) or desirable (desirability scored ≥ 1). ^d^Fold-change, proportion for odour detected divided by proportion for odour not detected. Data sourced from Wise et al. (2025).

Table S21: Impact of earthy odour detection to consumer impressions of cannabis.

| Variable | Earthy^a^ | N | Mean | StDev | FC^b^ | 2-sample t |
| --- | --- | --- | --- | --- | --- | --- |
| Interest | 0 | 465 | 7.804 | 1.9193 |  |  |
|  | 1 | 328 | 6.927 | 2.371 | 0.89 | 0.000 |
| Price | 0 | 465 | 12.516 | 3.647 |  |  |
|  | 1 | 328 | 11.463 | 4.168 | 0.92 | 0.000 |

^a^Detection of earthy odour No (0) or Yes (1), ^b^Fold-change, mean for odour detected divided by mean for odour not detected. Data sourced from Gilbert and DiVerdi (2018).

Table S22: Impact of earthy odour detection to consumer impressions of cannabis.

| Variable | earthy^a^ | N^b^ | Total^c^ | Proportion | 2-proportions test | FC^d^ |
| --- | --- | --- | --- | --- | --- | --- |
| High Quality | 0 | 52 | 13 | 0.25 | 0.597 | 0.75 |
|  | 1 | 32 | 6 | 0.19 |  |  |
| Desirable | 0 | 52 | 18 | 0.35 | 0.633 | 0.81 |
|  | 1 | 32 | 9 | 0.28 |  |  |

^a^Detection of earthy odour No (0) or Yes (1), ^b^Number of samples per odour detection category, ^c^Total samples assigned as high quality (quality scored ≥ 1) or desirable (desirability scored ≥ 1). ^d^Fold-change, proportion for odour detected divided by proportion for odour not detected. Data sourced from Wise et al. (2025).

Table S23: Ordinal logistic regression modelling desirability from citrus, clove, floral, and spicy odour detections

|  | Coefficient | P-value | Odds Ratio |
| --- | --- | --- | --- |
| Constant | -1.18 | < 0.001 | 0.31 |
| Citrus | 0.37 | 0.679 | 1.45 |
| Clove | 0.50 | 0.598 | 1.65 |
| Floral | 1.46 | 0.010 | 4.30 |
| Spicy | -0.68 | 0.378 | 0.50 |

Data sourced from Wise et al. (2025).

Table S24: Ordinal logistic regression modelling quality from citrus, clove, floral, and spicy odour detections

|  | Coefficient | P-value | Odds Ratio |
| --- | --- | --- | --- |
| Constant | -2.16 | < 0.001 | 0.12 |
| Citrus | 0.64 | 0.514 | 1.90 |
| Clove | -0.19 | 0.866 | 0.83 |
| Floral | 2.54 | < 0.001 | 12.65 |
| Spicy | -1.02 | 0.290 | 0.36 |

Data sourced from Wise et al. (2025).

Table S25. Summary of relationships between BC-associated odour changes and consumer perceptions

| BC/OD | FC^a^ | Relationship with liking/quality/other | Expected impact to perceived quality |
| --- | --- | --- | --- |
| BC1   - Lemon Peel | 1.47-fold | Positive | Positive |
| BC2   - Citrus | 1.03-fold | Positive | Positive |
| - Clove | 1.04-fold | Neutral | Neutral |
| - Floral | 1.02-fold | Positive | Positive |
| - Lemon | Present (absent in control and BC1) | Positive | Positive |
| - Lilac | 1.10-fold | Positive | Positive |
| - Orange | 1.06-fold | Positive | Positive |
| - Peely | 1.06-fold | Positive | Positive |
| - Spicy | 1.04-fold | Neutral | Neutral |
| - Thyme | Present (absent in control and BC1) | Neutral | Neutral |
| - Tropical | 0.83-fold | Positive | Negative |
| - Vegetable | 0.83-fold | Negative-neutral | Neutral-positive |

^a^Fold-change, mean for BC divided by mean for control.

References:

Gilbert, A. N., & DiVerdi, J. A. (2018). Consumer perceptions of strain differences in Cannabis aroma. *PLoS One*, *13*(2), e0192247.

Wise, K., Simovich, T., Gill, H., & Selby-Pham, J. (2025). Modulation of cannabis flower characteristics and THC through a biostimulant complex of molasses, *Aloe vera* extract, and fish-hydrolysate. *Folia Horticulturae*, *37*(3), 1-53. <https://doi.org/10.2478/fhort-2025-0022>
