# Supplementary material for: Comparison of two carbohydrate-based biostimulant complexes for their ability to enhance Cannabis sativa flower yield and quality
Source: Front Plant Sci. 2026 Jun 5;17:1842299. doi: 10.3389/fpls.2026.1842299 (PMC13278894; doi:10.3389/fpls.2026.1842299)
Supplement: Supplementary file 2 [file Table1.docx]

# Classify image pixels into Benavente-style colour categories

import os

import numpy as np

import pandas as pd

import matplotlib.image as mpimg

from math import atan2, degrees

from skimage import color

# ---- Output columns (Benavente categories) ----

OUTPUT_KEYS = [

"blue","white","pink","cyan","purple","black","grey","green","yellow","red","orange","brown"

]

# ---- Thresholds ----

# These numbers define how we separate achromatic colours (white/black/grey), highlights,

# and the chromatic wedges.

ACHRO_C_MAX = 4.0

WHITE_L_EDGE = 79.65

BLACK_L_EDGE = 28.28

WHITE_C_MAX = 9.0

HL_L_MIN = 97.0

HL_C_MAX = 16.0

# ---- Lightness bands ----

def lightness_band(L):

"""Return the lightness band (1–6) used to pick the hue wedges."""

if L < 20: return 1

elif L < 35: return 2

elif L < 50: return 3

elif L < 65: return 4

elif L < 80: return 5

else: return 6

def hue_deg(a, b):

"""Convert Lab a/b components to a 0–360 hue angle."""

h = degrees(atan2(b, a))

return h + 360.0 if h < 0 else h

def in_sector(h, left, right):

"""Check whether a hue lies inside a circular sector (left-inclusive, wrap-around)."""

if left <= right:

return (h >= left) and (h < right)

else:

return (h >= left) or (h < right)

def classify_pixel(L, a, b):

"""Map a single Lab pixel to a colour name from OUTPUT_KEYS."""

C = (a*a + b*b) ** 0.5

h = hue_deg(a, b)

# ---------- Achromatic ----------

if C <= ACHRO_C_MAX:

if L < BLACK_L_EDGE:

return "black"

elif L < WHITE_L_EDGE:

return "grey"

else:

return "white"

# ---------- Near/Highlight White ----------

if (L >= WHITE_L_EDGE and C <= WHITE_C_MAX) or (L >= HL_L_MIN and C <= HL_C_MAX):

return "white"

# ---------- Chromatic (Wedges) ----------

band = lightness_band(L)

if band == 1: # very dark (0–20)

if in_sector(h, 345, 15): return "pink"

if in_sector(h, 15, 35): return "red"

if in_sector(h, 35, 50): return "brown"

if in_sector(h, 50, 65): return "orange"

if in_sector(h, 65, 100): return "yellow"

if in_sector(h, 88, 195): return "green"

if in_sector(h, 180, 240): return "cyan"

if in_sector(h, 240, 290): return "blue"

if in_sector(h, 290, 345): return "purple"

return "grey"

elif band == 2: # dark (20–35)

if in_sector(h, 345, 15): return "pink"

if in_sector(h, 15, 35): return "red"

if in_sector(h, 35, 55): return "brown"

if in_sector(h, 55, 75): return "orange"

if in_sector(h, 75, 90): return "yellow"

if in_sector(h, 88, 195): return "green"

if in_sector(h, 180, 240): return "cyan"

if in_sector(h, 240, 290): return "blue"

if in_sector(h, 290, 345): return "purple"

return "grey"

elif band == 3: # mid-dark (35–50)

if in_sector(h, 345, 15): return "pink"

if in_sector(h, 15, 40): return "red"

if in_sector(h, 40, 70): return "orange"

if in_sector(h, 70, 95): return "yellow"

if in_sector(h, 88, 195): return "green"

if in_sector(h, 180, 240): return "cyan"

if in_sector(h, 240, 290): return "blue"

if in_sector(h, 290, 345): return "purple"

return "grey"

elif band == 4: # mid (50–65)

if in_sector(h, 345, 15): return "pink"

if in_sector(h, 15, 48): return "red"

if in_sector(h, 45, 75): return "orange"

if in_sector(h, 75, 100): return "yellow"

if in_sector(h, 100, 180): return "green"

if in_sector(h, 180, 240): return "cyan"

if in_sector(h, 240, 290): return "blue"

if in_sector(h, 290, 345): return "purple"

return "grey"

elif band == 5: # mid-light (65–80)

if in_sector(h, 345, 15): return "pink"

if in_sector(h, 15, 48): return "red"

if in_sector(h, 45, 75): return "orange"

if in_sector(h, 75, 102): return "yellow"

if in_sector(h, 102, 180): return "green"

if in_sector(h, 180, 240): return "cyan"

if in_sector(h, 240, 290): return "blue"

if in_sector(h, 290, 345): return "purple"

return "grey"

else: # band == 6 (80–100)

if in_sector(h, 345, 15): return "pink"

if in_sector(h, 15, 47): return "red"

if in_sector(h, 45, 75): return "orange"

if in_sector(h, 75, 105): return "yellow"

if in_sector(h, 105, 180): return "green"

if in_sector(h, 180, 240): return "cyan"

if in_sector(h, 240, 290): return "blue"

if in_sector(h, 290, 345): return "purple"

return "grey"

def analyse_image(img_rgb):

"""Convert an RGB image into Benavente category counts."""

# normalize to [0,1]; drop alpha if present

if img_rgb.dtype not in (np.float32, np.float64):

img_rgb = img_rgb.astype(np.float32) / 255.0

if img_rgb.shape[-1] == 4:

img_rgb = img_rgb[..., :3]

lab = color.rgb2lab(img_rgb)

# Lab separates lightness (L*) from chroma (a*, b*), which makes the thresholds easier.

L = lab[..., 0]; a = lab[..., 1]; b = lab[..., 2]

counts = {k: 0 for k in OUTPUT_KEYS} # prepare a counter for every output colour

H, W = L.shape

for i in range(H):

for j in range(W):

cat = classify_pixel(float(L[i, j]), float(a[i, j]), float(b[i, j]))

# Count how many pixels land in each category.

counts[cat] = counts.get(cat, 0) + 1

return counts

def main():

"""Analyse every image in the `cropped` folder and export the summary to Excel."""

df = pd.DataFrame()

folder = 'cropped'

for file in os.listdir(folder):

filepath = os.path.join(folder, file)

# Skip anything that is not a real image file (hidden files, directories, etc.).

if not os.path.isfile(filepath) or file.startswith('.DS_Store'):

continue

try:

img = mpimg.imread(filepath)

counts = analyse_image(img)

row = {k: counts.get(k, 0) for k in OUTPUT_KEYS}

df = pd.concat([df, pd.DataFrame(row, index=[file])])

except Exception as e:

# Keep going even if a single image fails to load or process.

print(f"⚠️ Skipped {file}: {e}")

df.to_excel('colours.xlsx')

print("✅ Analysis complete. Saved as colours.xlsx")

if __name__ == "__main__":

main()
